# Research Progress of Laser Cladding on the Surface of Titanium and Its Alloys

**DOI:** 10.3390/ma16083250

**Published:** 2023-04-20

**Authors:** Hui Zhao, Chaochao Zhao, Weixin Xie, Di Wu, Beining Du, Xingru Zhang, Min Wen, Rui Ma, Rui Li, Junke Jiao, Cheng Chang, Xingchen Yan, Liyuan Sheng

**Affiliations:** 1 Xi’an Key Laboratory of High Performance Oil and Gas Field Materials, School of Material Science and Engineering, Xi’an Shiyou University, Xi’an 710065, China; huier7921@126.com (H.Z.); chaoaooo@163.com (C.Z.); 2 Shenzhen Institute, Peking University, Shenzhen 518057, China; wudi@ier.org.cn (D.W.); bndu10s@alum.imr.ac.cn (B.D.); zxr_hit@126.com (X.Z.); wenmin1027@foxmail.com (M.W.); 3 Huizhou Port Customs, Huizhou 516081, China; xieweixin-0532@163.com; 4 PKU-HKUST Shen Zhen-Hong Kong Institution, Shenzhen 518057, China; marui200800@163.com (R.M.); lirui19930327@163.com (R.L.); 5 School of Mechanical Engineering, Yangzhou University, Yangzhou 225009, China; jiaojunke@yzu.edu.cn; 6 Institute of New Materials, Guangdong Academy of Sciences, National Engineering Laboratory of Modern Materials Surface Engineering Technology, Guangdong Provincial Key Laboratory of Modern Surface Engineering Technology, Guangzhou 510650, China; cheng.chang1993@hotmail.com (C.C.); yanxingchen@gdinm.com (X.Y.)

**Keywords:** titanium and its alloys, laser cladding, surface modification, process parameters, cladding materials, functional coatings, research status

## Abstract

Titanium (Ti) and its alloys have been widely employed in aeronautical, petrochemical, and medical fields owing to their fascinating advantages in terms of their mechanical properties, corrosion resistance, biocompatibility, and so on. However, Ti and its alloys face many challenges, if they work in severe or more complex environments. The surface is always the origin of failure for Ti and its alloys in workpieces, which influences performance degradation and service life. To improve the properties and function, surface modification becomes the common process for Ti and its alloys. The present article reviews the technology and development of laser cladding on Ti and its alloys, according to the cladding technology, cladding materials, and coating function. Generally, the laser cladding parameters and auxiliary technology could influence the temperature distribution and elements diffusion in the molten pool, which basically determines the microstructure and properties. The matrix and reinforced phases play an important role in laser cladding coating, which can increase the hardness, strength, wear resistance, oxidation resistance, corrosion resistance, biocompatibility, and so on. However, the excessive addition of reinforced phases or particles can deteriorate the ductility, and thus the balance between functional properties and basic properties should be considered during the design of the chemical composition of laser cladding coatings. In addition, the interface including the phase interface, layer interface, and substrate interface plays an important role in microstructure stability, thermal stability, chemical stability, and mechanical reliability. Therefore, the substrate state, the chemical composition of the laser cladding coating and substrate, the processing parameters, and the interface comprise the critical factors which influence the microstructure and properties of the laser cladding coating prepared. How to systematically optimize the influencing factors and obtain well-balanced performance are long-term research issues.

## 1. Introduction

Titanium (Ti) and its alloys have been commonly regarded as space metals, ocean metals, and biological metals in the 21st century due to their high specific strength, high fatigue resistance, high corrosion resistance, and excellent biocompatibility [1,2,3,4,5]. Thus, Ti and its alloys are widely used in aerospace, national defense, military, petrochemical, mechanical construction, and biomedical fields and other fields [6,7,8]. In 1940, Luxembourg scientist Knoll developed a method to reduce Ti from TiCl_4_ by magnesium, which became a common way for industrial manufacturing [7]. Ti is located in the fourth period and the IVB group in the periodic table of chemical elements with an atomic number of 22 and a density of 4.5 g/cm^3^. The melting point of Ti is 1668 °C, which is about 1000 °C higher than that of aluminum [9]. In general, Ti has a hexagonal closed-packed (HCP, α-Ti) lattice structure below 883 °C and then transforms to a body-centered cubic (BCC, β-Ti) lattice structure above 883 °C. Meanwhile, when some alloying elements are added to Ti, Ti alloys with different phase constituents are designed. These alloys have better properties compared with commercial pure Ti (CP-Ti), which promotes a wider application of Ti-based alloys. Generally, Ti alloys could be simply classified by the phase constituents, including α-Ti alloys, (α + β)-Ti alloys, β-Ti alloys, and shape-memory Ti alloys [10,11]. The α-Ti alloys are mainly composed of the α phase which is obtained by the α-stabilizers such as Al, O, and N. They have excellent castability and weldability, but their strength and plasticity are relatively low. In comparison, (α + β)-Ti alloys with a certain amount of α-stabilizers and β-stabilizers (such as V, Mo, and Ta) show a dual-phase microstructure (5–30% β phase) at room temperature. Compared with α-Ti alloys, these dual-phase alloys possess higher strength. In addition, they can be further strengthened by heat treatment [12,13]. β-Ti alloys with high amounts of β-stabilizers are mainly composed of a β phase. They display desirable corrosion resistance, higher strength, and better biocompatibility compared with other Ti alloys [14]. Shape memory Ti alloys have shaped memory effects and superelasticity, which derives from the reversible solid-state phase transformation between the higher temperature parent phase and lower temperature martensite phase. The TiNi alloy is a famous shape memory alloy and the first commercial Ti-based shape-memory alloy. Subsequently, many elements, such as Ag, Zr, Pd, and Hf, are added to the conventional TiNi alloys, which produces TiNiAg, TiNiZr, TiNiPd, and TiNiZrHf, respectively, and other Ti-based shape memory alloys [15,16]. Thus, the desirable properties of Ti and its alloys have been paid more attention. Ti and its alloys have become the most potential materials in more applications fields.

However, Ti and its alloys still have some disadvantages such as low hardness, an unsatisfied work hardening ratio, poor wear resistance, and so on [17,18]. For example, the microhardness of CP-Ti is in the range of 150–200 HV_1_ and the typical Ti-6Al-4V alloy has a microhardness of 290–375 HV_1_. Thus, Ti and its alloys show limited fatigue and wear resistance, which would easily result in component failure during its service. Moreover, the strength of Ti and its alloys would decrease sharply at higher temperatures (>600 °C), which indicates that Ti materials could not bear high loading in high temperature environments. In addition, as the choice of clinic implant, Ti and its alloys are inactive and could not induce tissue growth in vivo. However, the ion dissolution from Ti and its alloys or their wear debris could result in inflammation [19,20]. To conquer these disadvantages, many methods have been developed and surface modification has been thought of as the most convenient one.

### 1.1. Surface Modification for Ti and Its Alloys

#### 1.1.1. Buttering Welding

Buttering welding (BW) is a surface modification technology that can achieve metallurgical bonding between the substrate and cladding materials with an external heating source. Because of the metallurgical bonding, the BW cladding layer exhibits a relatively satisfactory service life [21]. Moreover, buttering welding has a wide scope of cladding materials, which can prepare wear-resistant layers, corrosion-resistant layers, and high-temperature-resistant layers on the substrate based on the requirements of workpieces [22]. The most commonly used cladding materials are Fe-based, Ni-based, Co-based, and Cu-based alloys [23]. Traditionally, buttering welding is performed by arc welding, submerged arc welding, and GMAW, which are relatively mature and widely applied. Recently, high-energy particle beam welding, tungsten inert gas welding (TIG), and plasma arc welding have been adopted to perform buttering welding [24]. Due to the abundance of cladding materials and external heating sources, the BW becomes one of the most convenient and economical methods to modify the substrate surface [25]. Rohan et al. [26] prepared a titanium matrix cermet (TMC) layer on the TC4 alloy surface with plasma transferred arc welding, which effectively improved the wear resistance of the TC4 alloy. Though BW has some advantages, it also possesses some shortcomings. The high heat output generates a large heat-affected zone, which influences the mechanical properties of the substrate greatly. Moreover, the big, remelted zone restricts its application in the workpieces with complicated shapes and precise dimensional tolerance. In addition, secondary machining is necessary for the BW modified surface.

#### 1.1.2. Arc Cladding

Different from the high thickness of buttering welding, arc cladding (AC) can produce a relatively thin modification layer by the arc heat source. Generally, argon (Ar) is always used as the protective gas during the arc cladding process, which can effectively isolate oxygen, avoiding the reaction of the cladding material with oxygen. Therefore, it also has similar advantages to buttering welding, such as low cost, simple process, easy operation, and large penetration depth of the cladding layer. Therefore, the macroscopic quality of the cladding layer surface prepared by arc cladding is relatively good, and the defects such as pores and slag inclusions can mostly be avoided [27,28]. Recently, a TiN-strengthened wear-resistant coating was prepared on a TC4 alloy surface by tungsten arc cladding [29]. With the increase in the TiN phase quantity in the cladding layer, the hardness of the cladding layer can reach up to 660 HV_1_, about 1.9 times of the matrix (350 HV_1_). Bao et al. [30] prepared a TiB-reinforced Ti-based composite coating on a TC4 alloy surface by the flux-cored wire and TIG, which exhibited that the TiB_2_ particles preferred to aggregate on the surface of the molten pool, contributing to the surface hardness. The as-prepared TiB-reinforced coating had higher hardness (571 HV_0.5_) and excellent wear resistance with minimum wear loss (14.10 mg). Huang et al. [31] added CO_2_ in Ar protective gas during the arc cladding on a TC4 alloy and revealed that the added CO_2_ promoted the solid solution of C and O and a subsequent reaction with Ti to form TiO_x_ and TiC reinforcements. The microhardness of the cladding layer is about 600 HV_1_ which is 1.7 times that of the Ar protective gas. However, the heat output of arc cladding is still relatively dispersed, which results in a heat-affected zone with a high width. In addition, the high heat output and rapid cooling could lead to cracks in the cladding layer [32].

#### 1.1.3. Plasma Cladding

Plasma cladding (PC) applies the high-energy plasma arc as a heat source to fabricate the cladding layer on the substrate surface, which has the characteristics of high energy concentration, fast heating speed, low cost, easy control, large area processing, high cladding efficiency, a high powder utilization rate, and a low dilution rate of the cladding layer. Due to the high energy concentration, the cladding layer could obtain good bonding strength with the substrate, which promotes its application in many lightweight metals [33,34]. Liu et al. [35] prepared a metal/ceramic composite cladding layer with the mixed powders of titanium iron and asphalt by plasma cladding, which is composed of fine TiC and Ti_2_O_3_. The microhardness of the composite coating was 1640 HV_0.2_ and had good wear resistance. Similarly, the TiB_2_-TiC-NiAl composite coating was prepared on the Q235 steel surface by plasma cladding with improved mechanical properties [36]. However, the crack is the defect that frequently appeared in the surface layer prepared by PC, which is mainly attributed to the wide heat affected zone and high residual internal stress.

#### 1.1.4. Electron Beam Cladding

Electron beam cladding (EBC) is another type of surface modification or repairing method with high-energy output. Unlike plasma cladding, the energy concentration of EBC is much higher and the size of the molten pool is smaller, which could obtain a cladding layer with low roughness [37]. Before EBC processing, a layer of alloy powders with a thickness of several microns to several millimeters would be preset on the surface of the substrate by spraying and then the cladding is performed by high-energy electron beam scanning [38]. Due to the high energy density, the cladding layer prepared by EBC has a dense structure and good bonding strength with the substrate. In addition, the high cladding rate and small molten pool decrease the heat-affected zone and residual stress [39,40,41]. The recent research prepared the (Ti,W)C_1−x_ composite coatings on the TC4 alloy surface by EBC with multi-phase and reinforcements [42]. The microhardness of the composite coating was up to 860 HV_1_, which improved the wear resistance greatly. Bataev et al. [43] prepared a Ti_3_Al layer on a CP-Ti plate by EBC, which increased the microhardness to 540–610 HV_1_ and obtained better sliding wear resistance and fixed abrasive friction. Lenivtseva et al. [44] fabricated a ceramic reinforced composite layer on a CP-Ti surface by EBC with 50 wt% TiC + 50 wt% CaF_2_ and 40 wt% Ti + 10 wt% graphite + 50 wt% CaF_2_ mixed powders. The results revealed that the powders with relative high melting points could be well dispersed in the cladding layer and have a good phase interface. Due to the well-dispersed TiC and graphite, the microhardness of the cladding could reach a maximum of 8 GPa and its wear rate was decreased to about 40% of the CP-Ti. Actually, the EBC also has some disadvantages. The EBC process needs a high vacuum environment and the electron beam is easily interfered with by stray electromagnetic fields, which restricts its application for components with a large size.

#### 1.1.5. Laser Cladding

Laser cladding (LC) is a rapidly developed surface modification method with combined advantages of EBC and AC, because of its high energy density and environment tolerability. Therefore, it could be used in high vacuum, gas shielding, or atmosphere environments. Due to the high energy density, LC could prepare the thin layer on the relatively complex structure surface from powder or wire feeding, while the adjusting of focal distance could change the depth of the molten pool and control the heat affected zone [45,46]. Combined with the small laser spot diameter, the thickness, surface roughness, and layer interface of the cladding layer could be optimized by parameter adjustment [47,48]. These advantages contribute to the formation of the cladding layer having metallurgical bonding with the substrate and good mechanical properties [49]. Lv et al. [50] prepared a hydroxyapatite (Ca_10_(PO_4_)_6_(OH)_2_, HA) composite layer on a CP-Ti surface by LC with mixed CaCO_3_ and CaHPO_4_ powders, which increased the biocompatibility obviously. The Ti_3_Al + TiB/Ti in situ composite layer was prepared on a TC alloy surface by LC from Ti and AlB_2_ powders [51]. The composite cladding layer increased the microhardness to 804 HV_0.3_ and decreased the wear rate to 12.5% of the TC4 substrate. Jiang et al. [52] fabricated a highly densified WC-Co composite layer on a TC4 alloy surface by LC, which demonstrated a well bonded interface. Moreover, the composite cladding layer obtained a microhardness as high as 1536 HV_0.5_ and a wear rate of 1.5 g/h.

**Figure 1:**
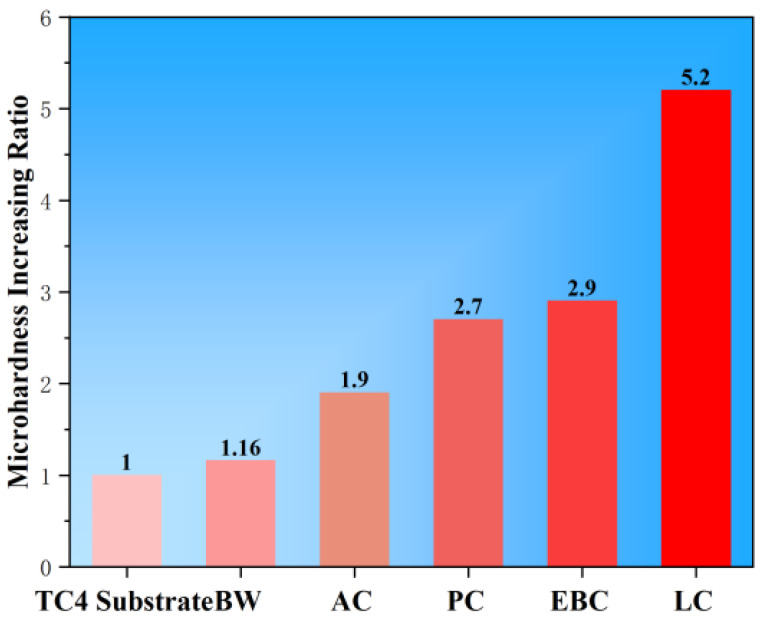
Microhardness enhancement ratio of cladding layers on a TC4 substrate fabricated by different methods and deepen color indicates the methods with hgih energy density. (BW: buttering welding; AC: arc cladding; PC: plasma cladding; EBC: electron beam cladding; LC: laser cladding).

Compared with well-developed surface modification technologies, it can be found that BW has a high dilution rate, which makes it difficult to obtain a thin and uniformly distributed cladding layer. Defects such as pores, slag inclusions, and arc crater cracks are prone to form, resulting in poor properties [53], while AC has shortcomings of shallow penetration depth, a slow cladding rate, and impurity from the tungsten electrode, which affects the cladding layer quality [54]. PC exerts redundant heat, which leads to great residual internal stress and deformation of the substrate [33,55]. For EBC, the requirement on the processing environment is high, which handicaps its wider application [56,57]. On the contrary, LC possesses the advantages of a high energy density, small molten pool, and high processing precision, which could optimize the cladding layer significantly [58,59,60]. As shown in Figure 1, the increasing ratio of the Y-axis microhardness represents the strengthening of multiples of the different coating microhardnesses relative to the TC4 substrate microhardness. The comparative analysis of the microhardness of the cladding layers fabricated by different surface modification methods demonstrates that the microhardness increases with the increase in energy density and the LC layer has the highest microhardness [12,26,36,45,46]. Besides the effect of microstructure and reinforcements, the improved remelting capability by the output energy density plays an important role. Table 1 illustrates a comparison of the various surface cladding processes.

Due to the obvious advantages of LC processing, it demonstrates application potential in many industry fields. However, as a kind of surface cladding method, the mechanical properties of the cladding layer could be influenced by the detailed processing parameters and cladding materials. Therefore, the recent research on the LC processed Ti and its alloys are reviewed in the present paper to demonstrate its prospects.

## 2. LC Processing

**Figure 2:**
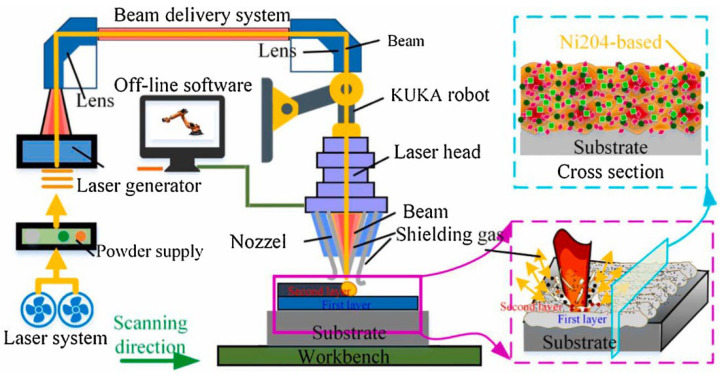
Schematic diagram of the LC system (reprinted from Ref. [61] with the kind permission of Elsevier).

LC processing is a process that remelts and merges the cladding materials and substrate surface simultaneously. The mechanical properties of the cladding layer are related to the LC parameters. Generally, the LC system is mainly composed of laser equipment, a motion control system, a powder feeding system, and protecting gas delivery, as shown in Figure 2 [61]. The laser equipment outputs the laser beam, which determines the laser type and output energy. The motion control system always employs the computer to control the robot, which can design the laser cladding route, laser cladding speed, laser spot size, and so on. The speed of powder feeding and the protecting gas shielding effect could be adjusted by the powder feeding system and protecting gas delivery. Therefore, many kinds of parameters could be set to optimize the LC processed layer.

Previous research [62] reveals that only a small proportion of laser power is used to melt the powders and most of the laser power is reflected off or used to remelt the substrate. Actually, the size of the molten pool is significantly influenced by the laser energy, which determines the height of the cladding layer [63]. To increase the molten pool size, either the output laser energy or the parameters should be adjusted. However, the higher output laser energy could lead to a deeper remelted substrate and a higher dilution ratio, which changes the mechanical properties of the substrate. Because the changing of laser scanning speed, focal distance, and the size of laser spot could be classified into the output of laser energy, the powder feeding method and auxiliary technology become the research issue.

### 2.1. LC Processing Technology

**Figure 3:**
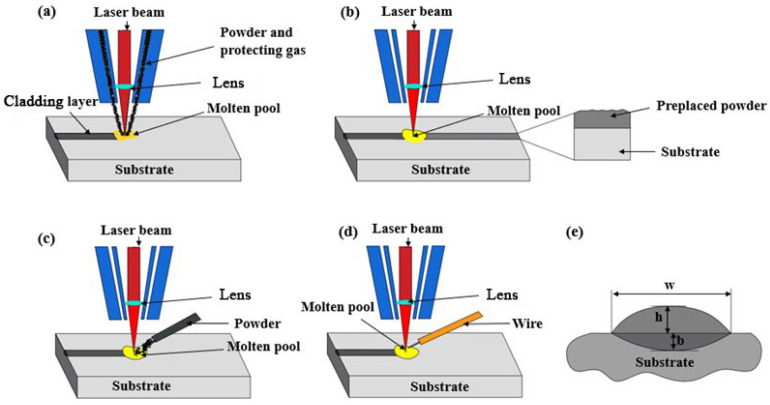
Schematic diagrams of the LC processing mode: (**a**) coaxial powder feeding method; (**b**) pre-placed powder method; (**c**) off-axis powder feeding method; (**d**) wire feeding method; (**e**) cross-sectional morphology of the cladding region.

Based on the powder feeding method, the LC processing mode mainly includes four kinds: coaxial powder feeding, pre-placed powder off-axis powder feeding, and wire feeding, as shown in Figure 3 [64]. For the coaxial powder feeding, the cladding powders are directly fed into the laser beam, and then the remelting and cladding are performed on the substrate surface simultaneously [65]. For the pre-placed powder, the cladding powders were spread on the substrate surface in advance and remelted by the subsequent laser beam scanning [66]. Off-axis feeding and wire feeding have similar processing, in which the powder and wire are fed into the molten pool and remelted by laser irradiation [67].

Coaxial powder feeding is the most commonly used powder feeding method, which could be applied to a structure with a complex shape. Because the powders are remelted on the substrate as they are sprayed, the cladding layer has a uniform thickness, small grain size, and good bonding strength with the substrate. Zhang et al. [68] prepared a TiC_x_/CrTi_4_-based composite layer on the surface of a TC4 alloy by coaxial powder feeding, which demonstrates a better appearance and fine microstructure with few cracks and pores. Due to the uniformly distributed TiC_x_ strengthening phase, the cladding layer exhibits significantly improved mechanical properties. The pre-placed powder method has more requirements on the shape of the substrate and the plane structure adopts this method preferentially. The mixed powders can be used in the pre-placed powder method to fabricate the composite cladding layer conventionally. Xu et al. [69] fabricated a TiC-particle-reinforced Ni-based gradient composite layer by LC with pre-placed ball-milled Ti, graphite, and Ni60A powders. The gradient composite layer improved the hardness and wear resistance of the substrate greatly. Off-axis powder feeding requires an additional pipeline to send the powders to the laser beam scanned region. Due to the short remelting time of powders, it is important to optimize parameters to obtain the well cladding layer. Corbin et al. [70] investigated the relationship between parameters and the quality of the cladding layer during the off-axis powder feeding assisted LC 6061 Al powder on the 6061-T6511 substrate surface. They demonstrated that the cladding layer height (h) increases with the increased ratio of powder feeding speed (F) and scanning speed (V), while cladding layer width (w) and molten layer depth (b) increase with the increased ratio of laser power (P) and scanning speed (V), as shown in Figure 3. Compared with traditional powder cladding, wire feeding cladding has the advantages of a good processing environment, high surface finish, and a high material utilization rate. However, the vibration of the wire during the feeding could seriously affect the cladding quality and processing stability.

Compared with the pre-placed powder feeding method, coaxial powder feeding, off-axis feeding, and wire feeding could be classified into synchronous cladding in which the feeding material is remelted simultaneously with the substrate surface. Because of the controlled heat input, the remelting, cladding, and solidification are finished in a short time, which could well control the cladding layer’s morphology. For the pre-placed powder feeding method, the laser irradiation melts powders first and then the substrate, which results in a bigger molten pool and influences the morphology of the cladding layer. In addition, the binder or moisture may lead to the formation of a pore in the cladding layer. As a result, the cladding layer obtained by the pre-placed powder method is not as uniform and dense as the coaxial powder feeding method. The off-axis powder feeding and wire feeding cladding methods could decrease the size of the molten pool but they are sensitive to the processing parameters. In contrast, the coaxial powder feeding process is stabler and more precise, because the remelting of powders by a laser beam and cladding with the molten substrate surfacealmost finish at the same time. The laser beam is not attenuated by the powder particles and the energy utilization rate is high, which benefits LC processing with a constant state. Therefore, the coaxial powder feeding method is the most widely used in the practical application of LC technology.

### 2.2. LC Processing Parameters

**Figure 4:**
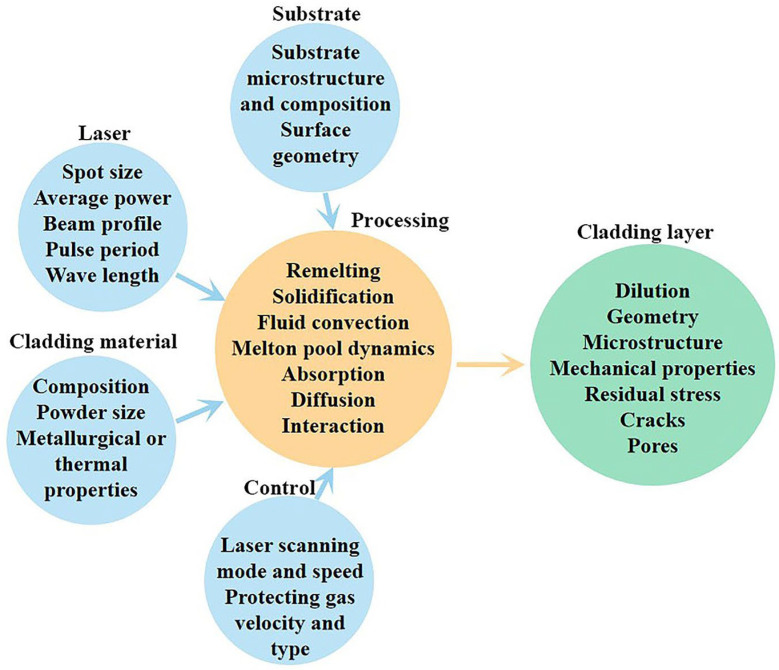
Schematic diagram showing the influence factors during LC processing.

For LC processing, its parameters are derived from the laser, cladding materials, motion robot, feeding, and gas delivery [71]. These parameters can exert an influence on the LC processing systematically and affect the remelting and solidification of the cladding materials and substrate, as shown in Figure 4. Generally, the morphology of the cladding layer is the focus that attracts the most attention, because it affects the quality of the modified substrate surface, while the metallurgical behavior of the cladding materials and substrate influences the microstructure and mechanical properties of the cladding layer. Therefore, the melt pool and its interaction with the substrate have been investigated based on the parameters.

For LC processing, the geometry of the cladding layer is related to the cladding region and remelting region, which could be defined as the dilution ratio in Equation (1) [71].

(1) $$\eta =\frac{S_{\mathrm{melt}}}{S_{\mathrm{melt}}+S_{\mathrm{clad}}}$$

where the *η* is the dilution ratio, *S_melt_* is the area of the molten substrate, and *S_clad_* is the area of the cladding layer.

Based on the description in Figure 3, the dilution ratio η is related to the height of the cladding layer and substrate melted layer. The increasing dilution ratio indicates that the height of the substrate melted layer is increased or the height of the cladding layer is decreased, which benefits the spreading of the cladding layer. Because the depth of the molten pool is highly dependent on the input energy, a higher laser energy density would increase the size of the molten pool. According to previous research [71,72], the laser energy density (*L_ed_*) could be the indicator of the amount of transferred energy from the laser, which is responsible for the melting of the cladding materials and substrate. The laser energy density could be defined as Equation (2).

(2) $$L_{\mathrm{ed}}=\frac{P}{{\mathrm{VD}}_{L}}({J/\mathrm{mm}}^{2})$$

where *P* refers to the laser power (W), *V* refers to the laser scanning speed (mm/s), and *D_L_* refers to the laser spot size (mm). This reveals that the laser energy density decreases with the increase in the laser scanning speed and spot size. Considering the small size of the molten pool and its thermal diffusion, the cooling rate of the molten pool would demonstrate the directionally solidified feature and relative coarse microstructure, which also influence the geometry of the cladding layer.

**Figure 5:**
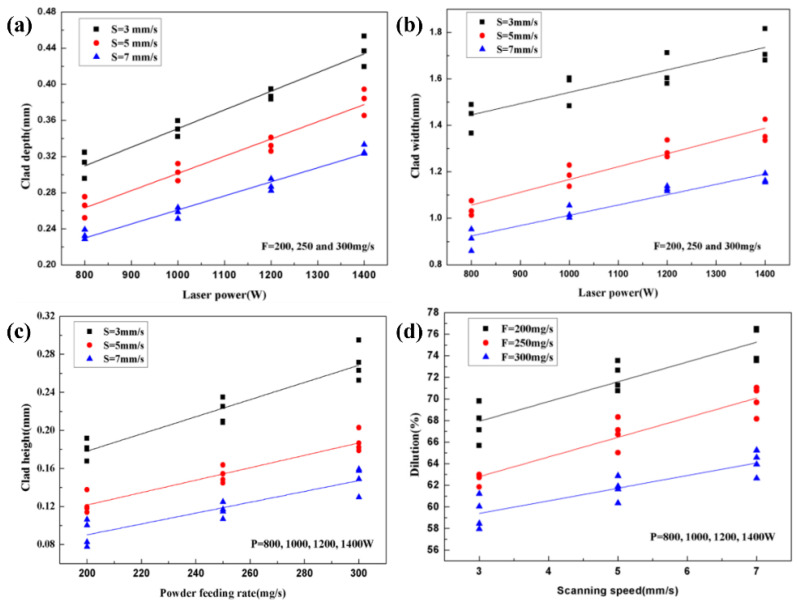
Influence of main processing parameters on geometrical characteristics of single-pass cladding: (**a**) the effect of the main processing parameter on the clad depth; (**b**) the effect of the main processing parameter on the clad width; (**c**) the effect of the main processing parameter on the clad height; (**d**) the effect of the main processing parameter on the dilution rate [73]. Reprinted with the kind permission of MDPI.

The investigation [73] on the LC ceramic-reinforced Ti-based powders reveals that the depth and width of the cladding layer increase with the laser power, while the height of the cladding layer and the dilution increase with the powder feeding rate and laser scanning speed, as shown in Figure 5. It can be seen that the depth and width of the cladding layer almost have a linear relationship with the laser power when the laser scanning speed is defined. When the powder feeding rate is specific, the dilution is almost linear with the laser scanning speed. This indicates that the laser power exerts a significant influence on the geometry of the cladding layer. Aghili et al. [74] remelted Cr_3_C_2_-NiCr mixed powders on the surface of titanium aluminide (TiAl) and revealed that the height of the cladding layer, wetting angle, and depth of the molten layer are proportional to the laser power and inversely proportional to the powder feeding rate and laser scanning speed.

Except for the typical analysis method based on energy density, there are still some other methods. Onwubolu et al. [75] fabricated a nickel-based alloy cladding layer on the steel surface and applied response surface methodology (RSM) to predict the geometry of the cladding layer. The results indicate that RSM could well predict the results and instruct the optimization of LC parameters. The research of Sun et al. [76] used RSM to analyze the parameters of laser cladding of a TC4 alloy and demonstrated that it could predict the geometry of the cladding layer, combined with experimental design and central composition design. Chen et al. [77] prepared a TiC cladding layer on a TC4 alloy surface and used a support vector machine (SVM) to analyze the results, which provided the optimized parameters for the following LC. Zhang et al. [78] set a novel process parameter optimization approach for laser cladding based on a multi-objective slime mold algorithm (MOSMA) and support vector regression (SVR), which obtained well-optimized LC process parameters.

Generally, the quality of the cladding layer by LC is obviously influenced by the parameters, and how to predict their impact is a meaningful research topic. The present investigation methods such as SRM, SVM, and SVR could help the parameters’ optimization. However, the analysis model is highly dependent on plenty of experimental data. In addition, the present analysis models mainly focus on hardness and wear, which is limited. Research on these analysis models should be carried out in the future to extend their prediction capability and demonstrate more mechanical properties.

### 2.3. Auxiliary Technology

**Figure 6:**
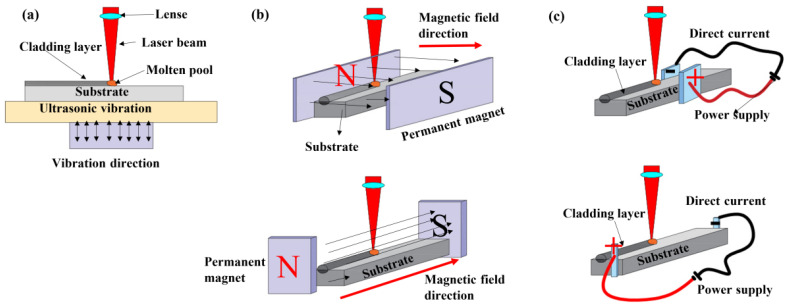
Schematic diagram of LC processing assisted with auxiliary technology: (**a**) ultrasonic vibration; (**b**) magnetic field treatment; (**c**) electric field treatment.

Based on LC parameters’ optimization, energy density is an important factor, which influences the size of the molten pool. The higher the energy density input, the bigger size of the molten pool. However, the increased laser energy density also increases the depth of the molten layer of the substrate, which is detrimental to the mechanical properties of the substrate. Moreover, the small size of the molten pool also results in rapid solidification and coarse dendrites during the LC process, which is harmful to the mechanical properties. Therefore, to optimize the microstructure of the molten pool and reduce the molten layer in the substrate, some kinds of auxiliary technologies have been developed, and some typical auxiliary technologies are shown in Figure 6.

Ultrasonic vibration (UV) is a useful technology that has been widely applied to optimize microstructures and decrease defects during the manufacturing of alloy components [79]. During laser cladding, UV could improve the fluidity of the molten pool and the microstructure during solidification. The previous research fabricated the Fe-Cr stainless steel part by UV-assisted laser cladding, which obtained the net shaping part with significantly decreased microcracks and pores, as shown in Figure 6a [80]. Research on the UV-assisted LC IN718 revealed that the cladding layer had a smaller grain size and the precipitates had been well refined, which improved the microhardness and quality of the cladding layer [81]. Moreover, the presence of UV during LC could redistribute the ceramic particles and decrease the friction coefficient significantly [82]. The well-improved microstructure and mechanical properties of the cladding layer by UV should be attributed to the inhibition of dendrite coarsening, which increases the melt fluidity and homogenizes the temperature distribution. Therefore, the molten pool has more time to undergo solidification and reduce the metallurgical defects. Chen et al. [83] revealed that UV-assisted LC decreased the internal tension stress by fragmenting dendrites and then alleviated the cracking around the interface. In conclusion, UV optimized the molten pool through the released sonic flow agitation, increasing the nucleation rate and improving the elemental distribution.

The external magnetic field and electronic field are always employed to improve the elemental diffusion during LC, as shown in Figure 6b,c. The magnetic field is produced by the permanent magnet or the electro-magnet, while the electronic field is produced by the electrodes fixed in a special position. The direction of the magnetic field or electronic field could be parallel or perpendicular to the direction of LC. The presence of a magnetic field could help the diffusion of elements and contribute to the microstructure optimization [84,85]. The magnetostriction effect decreases the thermal expansion and thermal stress, which decrease the crack sensitivity of the cladding layer by combining with well-reduced element segregation [86,87]. Moreover, the magnetic field could accelerate the flow in the molten pool and decrease the porosity, which is beneficial to the adhesion strength and wear properties of the cladding layer [88]. For electric-field-assisted laser cladding, the compound effect would be generated with thermal diffusion. Ouyang et al. [45] investigated the effect of the electrostatic field on the microstructure and mechanical properties of 316 L stainless steel prepared by laser cladding and revealed that the electrostatic field opposite the laser scanning direction promoted the elements’ diffusion and benefited directional solidification, but the electrostatic field consistent with the laser scanning direction restricted the elements’ diffusion and benefited ordered mushy solidification. Recent research has revealed that the electric field could increase the nucleation rate and refine the grain size, which improved the microhardness and corrosion resistance [89]. Actually, more research has optimized laser cladding processing by the mixture of electric and magnetic fields. Huo et al. [90] fabricated an In718/WC composite coating by electromagnetic-compound-field-assisted laser cladding and revealed that the induced downward Ampere force enhanced the Marangoni convection in the molten pool, which results in the uniformly distributed WC particles in the composite coating. In conclusion, either the electric field or the magnetic field influences the microstructure by the improvement of element diffusion or particle distribution. With the help of auxiliary technology, the laser cladding layer can obtain a better microstructure and mechanical properties. Auxiliary-technology-assisted LC would be the prospective process to perform the surface modification of Ti-based alloy.

## 3. LC Materials

During LC processing, the LC materials play an important role, because their properties directly determine the performance of the cladding layer. In order to obtain a cladding layer with better mechanical properties, it is necessary to select the appropriate cladding material. Except for the mechanical properties, the chemical composition and physical properties of the LC materials should be considered to meet the metallurgical requirements during LC processing [91]. Some principles could be set for the selection of LC materials [92]. Firstly, the cladding materials should have a similar thermal expansion coefficient to the substrate. Secondly, the melting temperature of the cladding materials should not be higher than that of the substrate. Thirdly, the cladding materials should have better wettability on the substrate. Based on the initial shape, the LC material could be divided into a powder, paste, wire, and bar. Actually, powders of LC materials have been widely applied in LC processing because of their better metallurgical molding capability [93,94]. According to the phase constituent, the powder LC materials can be mainly classified into the following categories: metal alloy powders, ceramic and ceramic composite powders, and rare earth (RE)-oxide-doped powders [95].

### 3.1. Metal Alloy Powders

Metal alloy powders are the most applied LC materials for the surface modification of Ti-based materials, because of their better metallurgical fusion during cladding. In addition, the metal-based interface between the cladding layer and substrate is beneficial to the adhesion strength [96]. The metal alloy powders for LC materials are mainly composed of self-fluxing alloy powders and high-entropy alloy powders, based on their physical or metallurgical properties.

#### 3.1.1. Self-Fluxing Alloy Powders

The self-fluxing alloy powder always contains the Si, B, and other elements which have a strong deoxidation effect [97,98]. Moreover, these elements help to decrease the melting temperature of all of the powders, which benefits self-melting during LC processing. In most cases, self-fluxing alloy powders are the first choice for LC materials. In addition, there are many kinds of self-fluxing alloy powders such as nickel-based, cobalt-based, and iron-based self-fluxing alloy powders, which could be applied for the surface modification of titanium alloys, magnesium alloys, and steel.

Ni-based self-fluxing alloy powders have the advantages of excellent wettability, good corrosion resistance, high-temperature self-lubrication, etc. They are suitable for cladding on structural parts with strict requirements on wear resistance, corrosion resistance, and fatigue resistance. The research on the NiCoCrAlY cladding layer on the TC4 substrate by LC demonstrated that the grain size in the cladding layer was influenced by the laser scanning speed, temperature gradient, and cooling speed [99]. With the optimization of LC parameters, the microstructure and mechanical properties of the NiCoCrAlY cladding layer were enhanced. Jeyaprakash et al. [100] compared the NiCrMoNb and NiCrBSiC cladding layers on the TC4 substrate by LC and exhibited that the refractory elements could help the wear resistance by the formation of the stiffness phase. Sun et al. [101] prepared a TiC/NiCrBSi composite coating by LC on the surface of TC4 and revealed that the solubility of TiC particles in the molten pool also increased with the increased laser power. The agglomeration of TiC particles refined the grain, which contributed to the dense cladding layer and improved the hardness.

Co-based alloy powders are another commonly used LC powder material with good wear and corrosion resistance at high-temperature [102,103]. During LC processing, the Co addition prefers to form the stiffness phases with the other refractory element, which is beneficial to the strength of the cladding layer [104]. Weng et al. [105] remelted the Co-based self-fluxing powders on a TC4 substrate by LC and demonstrated the formation of reinforcing phases such as CoTi, CoTi_2_, TiC, TiB_2_, TiB, Ti_5_Si_3,_ and so on. Furthermore, the occurence of ceramic particles in the cladding layer by Co-based self-fluxing powders would increase the wear resistance significantly by more than 10 times that of the substrate. However, the exceeding formation of ceramic particles in the cladding layer would coarsen the grain structure and be detrimental to the wear resistance.

Fe-based alloy powder materials have the advantages of low cost and good wear resistance. Usually, they are used in the LC modification of cast iron and low-carbon steel substrates. Compared with Ni-based and Co-based alloy powders, Fe-based alloy powders have some disadvantages such as poor self-fluxing properties, a high crack rate, poor oxidation resistance, etc. Therefore, the Fe-based alloy powders almost have no application in the LC modification of titanium and its alloys.

In the surface modification of Ti-based materials, the Ti-based self-fluxing powders are the most potential LC powder materials, because of their good metallurgical fusion. Ge et al. [106] prepared a Ti-Al-Nb alloy coating on the surface of TC4 by LC from mixed Ti, Al, and Nb powders. The microstructure analyses revealed that Ti_3_Al, AlNb_2_, and α-Ti are the main phases, which increased the hardness and wear resistance to 1.7 times and 2.9 times of the substrate, respectively. Because the conventional added elements of Si and B always induce the formation of a ceramic phase, this kind of LC material could be classified into ceramic composite powders. Actually, the self-fluxing alloy powders used for Ti-based alloy surface modification are mainly Co- or Ni-based powders (Table 2). In general, the self-fluxing alloy powders have similar properties to the substrate, which helps to obtain a better interface between the cladding layer and the substrate. The selection of the self-fluxing alloy powders is dependent on the working environment of the surface-modified parts.

#### 3.1.2. High-Entropy Alloy Powders

The high-entropy alloy (HEA) is a new type of material that is composed of five or more elements with similar molar ratios. Compared to conventional alloys, HEA has more advantages such as better corrosion resistance, high fatigue strength, higher oxidation resistance, good thermal stability, better yield strength, high hardness, good fracture toughness, good biocompatibility, and better abrasion resistance [49,113,114]. The improved properties of the HEA should be ascribed to the slow element diffusion, high entropy, cocktail effect, and lattice distortion [115,116,117]. Therefore, HEA powder has attracted much attention in the development of LC powder materials and many kinds of HEA powders have been developed, as shown in Table 3.

**Figure 7:**
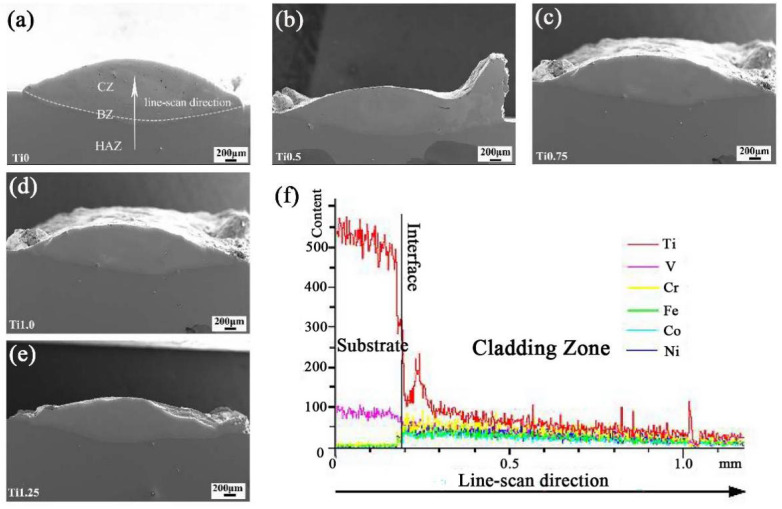
SEM images of the cross-section and line-scan result of the CrFeNi_2_V_0.5_Tix cladding layer with different Ti content: (**a**) Ti0; (**b**) Ti0.5; (**c**) Ti0.75; (**d**) Ti1.0; and (**e**) Ti1.25, and (**f**) line-scanning analysis results of Ti0 coatings (CZ: cladding zone; BZ: bonding zone; HAZ: heat-affected zone) [121]. Reprinted with the kind permission of MDPI.

A recent study fabricated an AlTiVMoNb HEA cladding layer on a TC4 surface and revealed that the HEA cladding layer increased the hardness and high-temperature oxidation resistance [120]. However, the element diffusion from the substrate induced the element distribution evolution [73]. Li et al. [121] prepared the CrFeNi_2_V_0.5_Tix HEA cladding layer on TC4 by LC and demonstrated that the Ti ratio influenced the morphology of the cladding layer, as shown in Figure 7. Figure 7a–e illustrated thes SEM images of the cross-section of the CrFeNi2V0.5Tix cladding layer with different Ti (0.25–1.25) content, and Figure 7f shows the line-scanning analysis results for the Ti0 coatings. With the increasing Ti content, the height of the cladding layer decreases, and the ratio of width and height increases gradually. In addition, the dilution by the remelted substrate increases the Ti content in the cladding layer. Furthermore, the interdiffusion between the cladding layer and substrate contributes to the improvement of the metallurgical bond. Interestingly, the hardness of the cladding layer remains unchanged when the Ti ratio in the HEA powder is below 1.0, but the hardness decreased significantly when the Ti ratio in the HEA powder was higher than 1.25, and the hardness decreased significantly. Considering the wear behavior, the HEA powders of CoCrFeNi_2_V_0.5_Ti_0.75_ could obtain the cladding layer with the best performance.

Though the HEA powders have some advantages, the multi-component composition feature always induces high dilution between the cladding layer and the Ti-based substrate, which influences the mechanical properties of the cladding layer and substrate. Therefore, more research is still needed to clarify the influence of interdiffusion on the microstructure stability and service life of the cladding layer and substrate, especially in a high-temperature environment. Actually, the improvement of the metal-based cladding layer is restricted by its intrinsic properties. Therefore, cladding materials with specific properties are developed.

### 3.2. Ceramics and Ceramic Composite Powders

Ceramics are a kind of material with a highly stable crystal structure and excellent tolerance to extreme environments, which contributes to its wide application [134,135]. For laser cladding, ceramic powders are always applied to improve the wear resistance, corrosion resistance, oxidation resistance, or hardness of the Ti-based materials [136,137]. Now, there are many kinds of ceramic powders developed for LC materials such as oxide, nitride, carbide, boride, and silicide. These ceramic powders could be used as cladding materials directly and fused with the remelted substrate surface. Due to their low wettability with metal, ceramic powders are mostly blended with metal to prepare the ceramic composite powders.

#### 3.2.1. Pure Ceramic Powders

**Figure 8:**
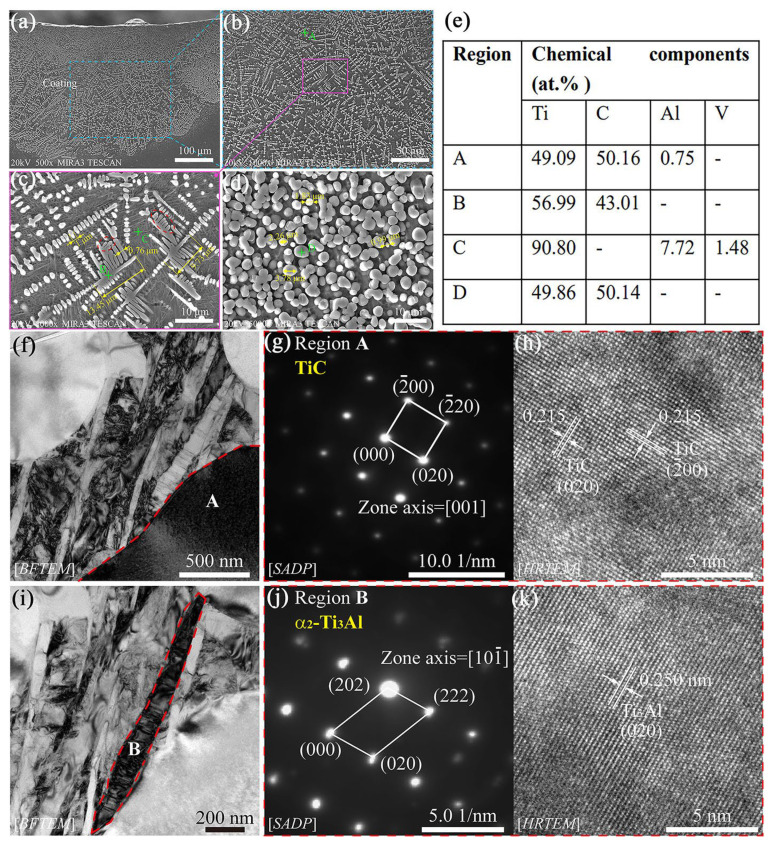
Microstructure of the TiC ceramic cladding layer and TEM analysis: (**a**) cross-sectional morphology; (**b**) low-magnification image and (**c**) high-magnification image of TiC dendrites; (**d**) high-magnification image of TiC granular grains; (**e**) EDS analysis results of TiC ceramic coating (A,B,C,D are the selected areas in (**b**–**d**)); (**f**) BFTEM image of the TiC phase and its corresponding; (**g**,**h**) SADP and HRTEM images of the TiC phase; (**i**) BFTEM image of the α_2_-Ti_3_Al phase and its corresponding; (**j**,**k**) SADP and HRTEM images of the α_2_-Ti_3_Al phase (Reprinted from Ref. [138] with the kind permission of Elsevier).

During LC processing, the pure ceramic powders need more energy to fuse with the Ti-based substrate, due to its high melting point. In addition, most ceramic powders prefer to aggregate on the surface of a molten pool, due to their relatively low density. Therefore, auxiliary technology or reaction synthesis is usually designed to obtain the well distributed ceramic cladding layer. Recent studies [138,139] prepared a TiC-ceramic-strengthened cladding layer on a TC4 substrate surface by LC from nano TiC powders, as shown in Figure 8. The nano TiC powders were remelted and recrystallized during LC processing, which promoted the TiC dendrites in the cladding layer. Even though the TiC is recrystallized, the TiC dendrites are still segregated in the cladding surface. The remelted substrate surface diluted the TiC ceramic, while the precipitation of TiC induced the formation of the α_2_-Ti_3_Al phase, as shown in Figure 8f–k. With the strengthening of the TiC ceramic cladding layer, the microhardness and fracture toughness of the substrate were improved without any influence on biocompatibility. Chen et al. [102] fabricated a TiC/TiB ceramic cladding layer on a TC4 substrate surface from in situ synthesis by LC, which demonstrated that the multi-ceramic phases could be obtained simultaneously. In addition, the mechanical properties of the substrate surface were enhanced significantly. Tian et al. [140] prepared a composite cladding layer on a TC4 substrate surface from C and Si mixed powders and showed the formation of TiC and Ti_5_Si_3_ ceramic phases. The refined TiC and Ti_5_Si_3_ ceramic phase in the cladding layer increased the hardness to 1500 HV_0.1_ and decreased the wear resistance to a quarter of the substrate. Previously, the TiN had been fabricated on the Ti-based alloy by laser nitriding, which improves the corrosion resistance obviously [141].

Although the ceramic cladding layer could the improve hardness, wear resistance, and corrosion resistance, the significant difference in the thermal expansion coefficient and elastic modulus between the ceramic and Ti-based alloys always induces great internal stress, which prefers to result in cracks and even interface delamination [142]. In addition, the formation of a ceramic phase in the molten pool would decrease the fluidity and promote the solidification defects [143]. Such disadvantages restrict the application of pure ceramic powders in the surface modification of Ti-based alloys by LC.

#### 3.2.2. Ceramic/Metal Composite Powders

Considering the disadvantages of pure ceramic cladding powders, ceramic/metal composite powders are the cladding materials with the most potential for LC processing. Especially for the ceramic cladding powders with the addition of Ti-based alloying powders, they could make full use of the strengthening effect of ceramic powders and the fusion effect of Ti-based alloying powders, which benefits the metallurgical bond between the cladding layer and substrate. Moreover, the better weldability and ductility of the metal powders would enhance the toughness, wear behavior, and so on. Therefore, ceramic/metal composite powders have become the research focus for the surface modification of Ti-based materials. Until now, many kinds of ceramic/metal composite powders have been developed such as Ti-based ceramic composite powders, Ni-based ceramic composite powders, and Co-based ceramic composite powders.

The research [8,61,107,110,111,144,145,146,147,148,149,150,151,152,153,154,155,156,157,158,159,160,161,162,163,164,165,166] on the ceramic/metal composite powders for LC processing reveals that in situ and extrinsic strengthening ceramic phases have been widely applied in the development of ceramic/metal composite powders for LC processing, as shown in Table 4. For the Ti-based alloy substrate, the Ti-based ceramic strengthening phases are always preferred and cooperate with the Ti-based alloy powders, which contributes to obtaining a cladding layer with excellent wear resistance. Moreover, the B_4_C ceramic powder is added to the composite powders to achieve the synthesized Ti-based phase or other ceramic phases in the cladding layer. However, the WC, h-BN, and ZrO_2_ ceramic particles with ultra-high stiffness are also applied in the ceramic/metal composite powders to improve the hardness and wear resistance of the cladding layer. In general, the present research on the surface modification of Ti-based alloy substrates by ceramic/metal composite powders mainly focuses on hardness, wear resistance, and oxidation resistance.

Actually, the mechanical properties of the cladding layer fabricated by the ceramic/metal composite powders are closely related to the powders’ morphology, ceramic/metal ratio, ceramic distribution, fusion state of the composite powders, and dilution effect, etc. The systematical optimization of these factors would benefit the microstructure of the cladding layer, which enhances the mechanical properties of the cladding layer. Based on the chemical composition of the metal additive, the Ti-based composite powders with ceramic particles have the best metallurgical bond and excellent properties at room temperature. The Co- and Ni-based composite powders with ceramic particles have better properties at high temperatures. In conclusion, ceramic/metal composites are one of the most widely used and studied cladding materials for the surface modification of Ti-based alloy substrates by LC processing.

#### 3.2.3. Bioceramic Composite Powders

Due to the rapid formation of TiO_2_ passivation film on the surface, Ti-based alloys possess excellent corrosion resistance, which benefits their biocompatibility. Therefore, Ti-based alloys have been widely applied to fabricate biomedical implants which are used in orthopedics and stomatology, etc. However, the formed TiO_2_ passivation film has no bioactive function and cannot realize the molecular association with soft or hard tissue. In addition, Ti-based alloys always have an ion releasing effect that may be detrimental to the biocompatibility. To improve the biocompatibility of Ti-based alloy implants and promote their synostosis, the surface modification on the Ti-based alloy has been investigated thoroughly [167]. For LC processing, bioceramic composite powders are a hot research topic and have been studied widely. According to their functional features, the bioceramic powders are mainly divided into bioactive ceramic powders and bioinert ceramic powders. The bioactive ceramic powders can increase biological activity and contribute to the capability of synostosis, such as ceramic glass and hydroxyapatite (HA) [168,169], while the bio-inert ceramic powders mainly act as the shielding layer for the Ti-based alloy to avoid ion release, such as TiN or ZrO_2_ ceramic [134].

**Figure 9:**
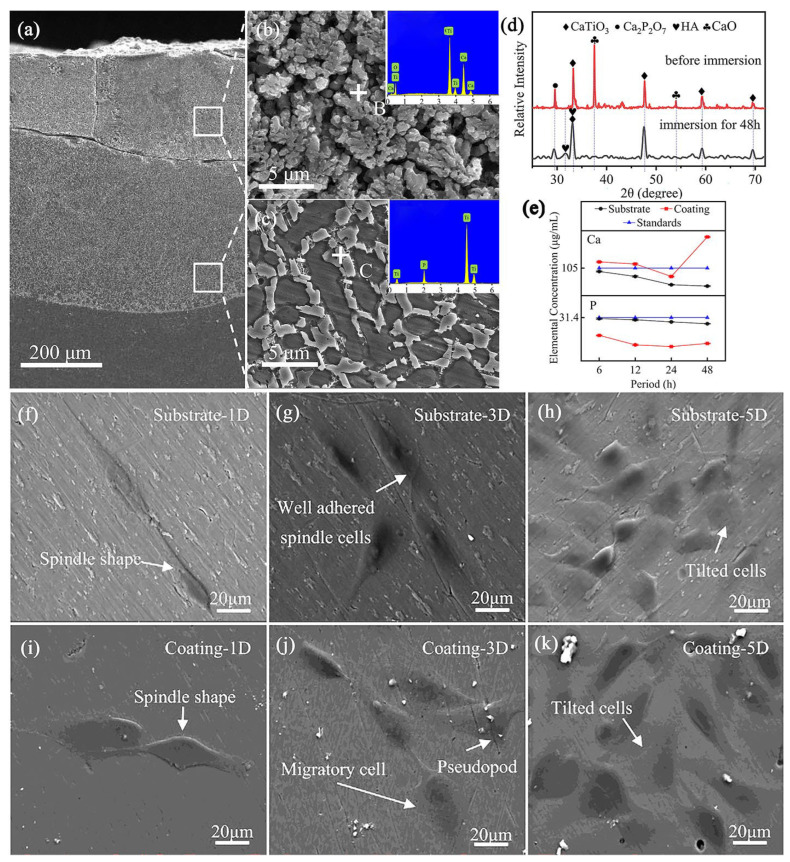
(**a**) The macrograph of the Ca/P ceramic cladding layer on the TC4 substrate; (**b**) microstructure and the EDS results at point B of the cladding layer; (**c**) microstructure and the EDS results at point C of the transition layer; (**d**) XRD patterns of the cladding layer with and without immersion; (**e**) Ca and P concentrations in the immersion solution with different specimens and time; (**f**–**h**) morphology of the cultured MG-63 cells on the TC4 substrate for 1, 3, and 5 days; (**i**–**k**) morphology of the cultured MG-63 cells on the Ca/P cladding layer for 1, 3, and 5 days [171]. Reprinted with the kind permission of MDPI.

Recently, many researchers [107,167,170] have performed investigations on the HA bioceramic composite layer prepared by LC on a TC4 alloy substrate, which demonstrated greatly improved biocompatibility. Liu et al. [171] fabricated a Ca/P bioceramic layer on a TC4 alloy substrate from hydroxyapatite powder by LC processing. The bioceramic-based composite layer was mainly composed of α-Ti, Ti_3_P, TiO, CaO, CaTiO_3_, and β-Ca_3_(PO_4_)_2_ phases and had no cracks or pores inside (Figure 9a–d). During the simulated body solution immersion test, the Ca/P bioceramic cladding layer released more Ca and P ions (Figure 9e), which benefited the formation of HA on the surface and the proliferation of MG-63 cells, as Figure 9f–k shows the morphology of the MG-63 cells cultured on the substrate. Compared with the TC4 alloy substrate, the Ca/P cladding layer improves the biocompatibility obviously.

Li et al. prepared a CaO-SiO_2_-MgO bioceramic composite coating on a TC4 alloy surface to improve its biological activity and revealed that the formation of CaTiO_3_ refined the microstructure of the ceramic layer and contributed to the performance [172]. Zhu et al. Fabricated a calcium phosphate bioceramic coating on the surface of a titanium alloy by LC, which demonstrated the formation of bioactive phases of β-tricalcium phosphate (β-TCP) and hydroxyapatite (HA) in the ceramic layer [173]. In fact, the osteoclasts promoted the dissolving of the bioceramic phase and resulted in small pores on the surface, which helped the proliferation of osteoblasts and benefited the entire biocompatibility. Among the bioceramic materials, HA is the most applied one, because of its bioactive advantage during osseointegration. Therefore, HA or HA-based composites have been considered as the candidates with the most potential for the surface modification of Ti-based implants, such as intramedullary nails, fixation plates, screws, etc.

For biomedical implants, the surface modification is important to enhance their biocompatibility, but the reasonable selection of cladding materials is the crucial factor [174]. An ideal bioceramic cladding material should have a bioactive effect and fuse well with the substrate. Due to the obviously different thermal expansion coefficients between the bioceramics- and Ti-based alloy substrates, thermal stress is always generated along the interface and leads to cracks. Wollastonite and wollastonite-based silicate powders are developed for Ti-based alloy surface modification, because of their osteoconductive features and similar thermal expansion to Ti-based alloy [175]. In addition, the bioceramic particle strengthened alloy powders become the most selected choice for the surface modification of Ti-based biomedical alloy by LC processing. However, the surface properties are related to the ratio of bioceramics, powders shape, substrate state, LC parameters, and so on. Therefore, the properties of the cladding layer from bioceramics-based powders are not stable, and more investigations should be performed to reveal the internal mechanisms.

### 3.3. Rare-Earth-Oxide-Doped Powders

The cracks formed in the cladding layer are the main defects generated during LC processing. To eliminate such defects, post-heat treatment is mostly applied but the treatment time is long and difficult to perform on large workpieces. Therefore, microstructure control during the LC process becomes a helpful strategy to restrict the crystal coarsening and decrease thermal stress. The rare earth (RE) elements have the effect to refine the microstructure and increase mechanical properties [176,177,178]. During LC processing, the added RE oxides prefer to arrest the oxygen or impurities in the molted pool and act as the nucleus and pinning particles [179]. The optimization of solidification behavior and microstructure would contribute to the surface morphology, mechanical properties, and wear behavior.

Previous research [180] added nano Y_2_O_3_ particles in TiC/Ni composite powders and prepared the cladding layer on a TC4 alloy substrate. The microstructure analyses revealed that the Y_2_O_3_ dispersedly distributed in the cladding layer and promoted the relatively uniform distribution of TiC particles, which increased the microhardness to 1380 HV_1_. Zhang et al. [181] added different Y_2_O_3_ in TC4-Ni45-Co-WC composite powders and prepared the cladding layer on a TC4 alloy surface, which revealed that the reasonable Y_2_O_3_ content could almost eliminate the cracks. Such an improvement was mainly ascribed to the higher supercooling resulting from the existence of Y_2_O_3_ and the wear properties are benefited by the microstructure evolution. Gong et al. [182] added CeO_2_ in a Ni60A powder to prepare the cladding layer on the TC4 alloy substrate and found that the added CeO_2_ promoted the absorption of energy and increased the fluidity of the molten pool. The improvement of melt reduced the crack sensitivity of the cladding layer and increased the hardness and wear resistance. Liu et al. [183] fabricated a γ/Cr_7_C_3_/TiC composite cladding layer on a TiAl alloy substrate by LC and demonstrated that the addition of La_2_O_3_ could refine and purify the cladding layer and decrease the volume fraction of primary blocky Cr_7_C_3_. In addition, the dilution effect was weakened and the microhardness of the cladding layer increased. Research on a CeO_2_-doped Ni-based composite cladding layer revealed that the appropriate CeO_2_ content could improve the corrosion resistance, but more CeO_2_ content would result in a reduction [184]. Based on the properties required on the surface, many kinds of RE-oxide-doped composite powders have been developed, as shown in Table 5.

During LC processing, the solidification rate and impurity influence the microstructure and mechanical properties greatly. Especially the ceramic- or refractory metal- based powders, they decrease the fluidity of melt and the involved impurities enhance the effect further [198]. The addition of RE oxides could make full use of their reactive surface and arrest the impurity, which purifies the melt and benefits the fluidity. However, the RE oxides also prefer to aggregate together and excessive addition would lead to segregation and microstructure coarsening [199]. In addition, the presence of RE oxides in the cladding layer would affect the microstructure during the heat treatment. Therefore, the balanced RE oxide addition is the crucial factor for the RE-oxide-doped powders. In addition, the LC parameters should be adjusted according to the composition of the RE-oxide-doped cladding powders.

In summary, the performance of the cladding layer obtained by LC is related to the powders primarily. The morphology, chemical composition, and the size of the powders are the main features that influence the microstructure and mechanical properties obviously. Based on the property requirements, reasonable powders should be selected to obtain the cladding layer. In turn, the selected powders determine the LC parameters. It is a crucial issue to obtain the cladding layer with reasonable dilution, low internal stress, and a fine and uniform microstructure. Although some progress has been achieved in the development of LC powders, it is still a challenge to quantitatively design the content of the powder based on the required performance on the cladding layer. More research is needed to on LC powders for their diversification, serialization, and standardization.

## 4. Functional Coatings

Though Ti and its alloys have relatively excellent performance, severe working environments always exert different impacts on the components. Therefore, the Ti and its alloy components have to endure one or more influencing factors such as wear, high-temperature oxidation, and corrosion. To obtain more capabilities, surface modification based on LC processing could be applied and a series of corresponding functional coatings were developed, including wear-resistant coating, corrosion-resistant coating, high-temperature oxidation-resistant coating, biological coating, and so on [200].

### 4.1. Wear-Resistant Coatings

Ti and its alloy components are prone to failure in complex service environments due to their low surface hardness and weak wear resistance. By selecting appropriate cladding materials and LC processing parameters, the hardness and wear resistance of Ti and its alloys surface could be improved significantly. Generally, there are many kinds of methods to improve surface performance, such as fine grain strengthening, solid solution strengthening, and dispersion strengthening. Actually, the dispersion strengthening with the stiffness phase is the most potential one, because of its obvious strengthening effect. Moreover, this kind of strengthening method is also convenient to realize by the reinforced phases such as ceramic or intermetallic compounds [201,202]. Based on the content of the reinforced phase, the wear-resistant coating could be mainly divided into the metal matrix ceramic composite coatings and intermetallic compound coatings, whose strengthening effect is related to the type, quantity, distribution, and interface of the reinforcing phase [203].

**Figure 10:**
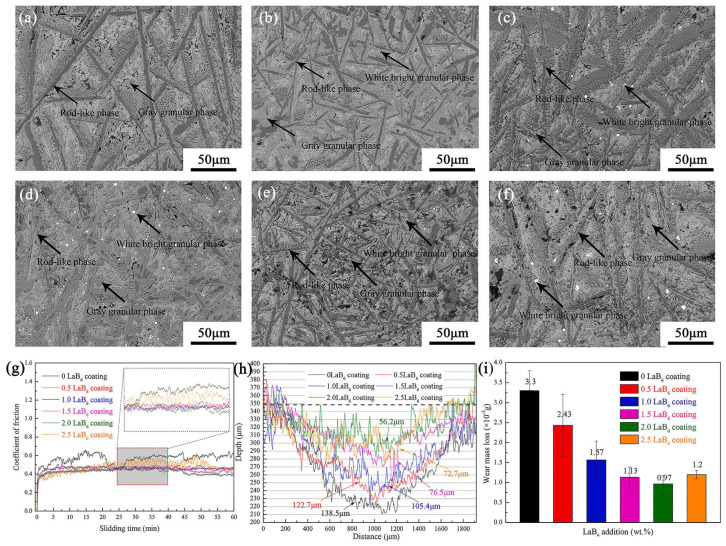
SEM images of the microstructure in the upper area of each coating: (**a**) without LaB_6_ addition, (**b**) 0.5 LaB_6_ addition, (**c**) 1.0 LaB_6_ addition, (**d**) 1.5 LaB_6_ addition, (**e**) 2.0 La_6_ addition, (**f**) 2.5 LaB_6_ addition, (**g**) friction coefficient of the cladding coatings with different LaB_6_ additions, (**h**) wear depth of the cladding coatings with different LaB_6_ additions, and (**i**) wear loss of the cladding coatings with different LaB_6_ additions (Reprinted from Ref. [146] with the kind permission of Elsevier).

Due to the high stability and stiffness of the ceramic, more LC composite coatings select the ceramic, such as TiC, TiB, TiB_2_, TiN, SiC, and WC, as the reinforced phase. Recent studies [193,194,195,196] prepared TiB_2_ particles or TiB-short-fiber-reinforced titanium-based composite coatings on a titanium substrate surface with excellent wear-resistant properties. Due to the nucleation effect of the ceramic particles, the corresponding composite coatings always have a fine grain structure, which increases the microhardness and wear resistance further. Therefore, the TiB_2_- or TiB-reinforced composite coating increased the wear resistance twice as much as the titanium substrate. However, the excessive ceramic addition would lead to its aggregation and coarsening. To avoid the coarsening of the single phase, a recent study adopted the in situ formation of a multi-ceramic reinforced phase in the LC coating by the addition of LaB_6_ and B_4_C powders [146]. The results revealed that the amount of TiC, TiB, and TiB_2_ phases increased with the increased LaB_6_ and B_4_C content, as shown in Figure 10a–f. Moreover, the addition of LaB_6_ resulted in the formation of La_2_O_3_ along the grain boundary of TiB_2_, which restrained its growth and promoted the thinning of the TiB_2_ rod-like phase. The evolution of multi-ceramic phase morphology induced an increase in microhardness but a decrease in wear resistance, as shown in Figure 10g which shows the coefficients of friction curves. Compared with the LC coating without LaB_6_, the wear loss of the best multi-ceramic phase LC coating decreased by about 71%, as shown in Figure 10h,i which shows the section profiles of the wear track and the wear mass losses of the coatings with different amounts of LaB_6_ addition.

Though the ceramic composite LC coating could improve the wear resistance of the titanium-based materials, the high brittleness of the ceramic phase and its interfacial mismatching with the matrix are prone to induce microcracks and failure [204]. The intermetallic compound has a metallic constituent and a long-range ordered crystal structure, which ensures its high strength and thermal stability [202,205,206]. Therefore, intermetallic compounds were introduced into the LC composite coating on titanium or its alloys’ surface modification. Usually, it is preferred for Ti-Al-, Ti-Ni-, and Ti-Co-based intermetallic compounds to be chosen as the reinforced phase, such as TiAl, Ti_3_Al, Ti_5_Si_3_, Ti_2_Ni, CoTi_2_, CoTi, AlTi_2_, and AlCo_5_, etc. In order to improve the wear resistance of the TC4 alloy, the TiAl, CoTi_2,_ and Ti_5_Si_3_ phase strengthened composite coatings were fabricated, which decreased the friction coefficient and wear rate greatly [207]. In addition, some intermetallic compounds modified the matrix and could produce a self-lubricating behavior at high temperatures, which could be used to design the special component. More studies have made full use of intermetallic compounds and ceramic phase to design LC coatings with balanced properties. Feng et al. [51] prepared a (Ti_3_Al + TiB)/Ti composite coating on a TC4 alloy by LC and obtained a good metallurgical bond between the coating and the substrate. The in situ formed Ti_3_Al and TiB phases interacted with each other, which refined the grain of the LC coating and increased its hardness to 2.3 times that of the matrix. The (Ti_3_Al + TiB)/Ti composite coating prepared by LC changed the wear behavior from the adhesive wear of TC4 alloy to the present micro-cutting and brittle peeling, And it decreased the friction coefficient and wear loss to 50% and 12.5% of the substrate, respectively.

Comparatively, the titanium matrix of the coating still has some disadvantages, such as low strength and wear resistance. Then, some studies have introduced the heterogeneous alloy coating on the titanium alloy surface. Wang et al. [208] fabricated a Ni-based composite coating on a TA2 pure titanium substrate surface from Ni-Trihaloy700 alloy powders by LC, which mainly comprised the Laves phase and γ matrix. Combined with the fine grain structure, the solid solution strengthening effect by Cr and Mo alloying elements increased the strength of the coating and its microhardness was about five times that of the substrate. Lu et al. [209] prepared a different hexagonal boron nitride (h-BN)-doped Ni60 composite coating on a TC4 alloy substrate by LC and revealed that the combination of h-BN, TiC, and TiB_2_ strengthening particles could improve the microhardness to 1155.32 HV_0.2_ which is about three times that of the substrate (about 370 HV_0.2_), and the wear resistance was improved simultaneously.

Based on the recent research on wear resistance coatings prepared by LC, it can be summarized that the strengthening phase and matrix microstructure play an important role by increasing the hardness and restricting wear scratching. Especially for the coating with the self-lubricating requirement, the phase constituent would exert great influence. In addition, the quality and bonding strength of the LC coating also contributes much to the wear resistance. Due to the different advantages of ceramic composite coating, intermetallic compound composite coating, and heterogeneous alloy coating, they could be utilized in different environments.

### 4.2. Corrosion Resistant Coatings

Generally, Ti and its alloys easily react with oxygen to form a dense TiO_2_ oxide film on the surface, which makes them have good corrosion resistance [210]. However, in some extreme service conditions (such as high temperature, erosion–corrosion, or high salt environment), the TiO_2_ oxide films would be destroyed, which exposes the substrate to the service environment [211]. The corrosion medium would accelerate the corrosion rate on the substrate. Therefore, the failure of the surface protecting layer is detrimental to the long-term service and safety of Ti and its alloy components, which handicaps their application in many fields. To meet the requirements of the extreme service, surface modifications are always applied to form the corrosion resistant coating and improve the surface properties [142]. Especially for the component with impact or other loadings, the LC coating on the Ti and its alloy component would be more valuable, because of its better bonding strength.

**Figure 11:**
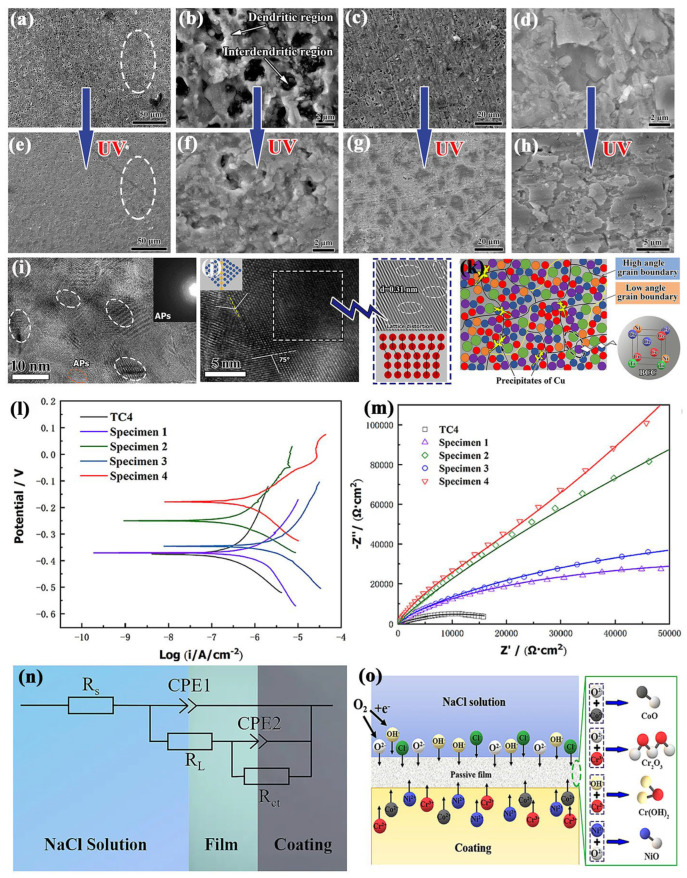
Microstructure and electrochemical performance of the CoCrNi-medium-entropy-alloy-based coating on TC4 substrates: (**a**,**b**) low laser power fabricated coating (specimen 1); (**c**,**d**) high laser power fabricated coating (specimen 3); (**e**,**f**) low laser power fabricated coating with UV (specimen 2); (**g**,**h**) high laser power fabricated coating with UV (specimen 4), (**i**,**j**) HRTEM image showing the substructure and crystal defects (the white dotted circles indicating formed dislocations); (**k**) schematic diagram of atomic structure; (**l**) Tafel polarization curves; (**m**) Nyquist plots in 3.5 wt% NaCl solutione; (**n**) equivalent circuit diagram, and (**o**) schematic diagram of the corrosion mechanismm (Reprinted from Ref. [216] with the kind permission of Elsevier).

Considering the interfacial bonding and corrosion resistance, metal- and ceramic-based coatings are the main choice for the corrosion-resistant coating of Ti and its alloys fabricated by LC. For the metal-based corrosion resistant coating, the Ni, Cr, Co, Al, and rare earth elements are the preferred elements that could form the phases with high corrosion resistance. In addition, the solid solution effect and dispersed precipitates help to improve the strength of the LC coating and benefit the erosion–corrosion resistance. Recently, many studies have investigated Ni-Co-Cr based cladding coatings on Ti-based substrates to obtain excellent corrosion resistance [212,213,214,215]. The results indicated that a good metallurgical bond could be realized between the cladding coating and substrate without any cracks and pores by the optimization of parameters. Compared with the Ti-based substrate, the corrosion voltage and current of the cladding coating covered Ti-based substrate decreased significantly in different corrosion environments (such as 3.5% NaCl solution, simulated body fluid), especially the corrosion current. Hu et al. [215] prepared Ni-based alloy (1.0 wt% C, 16.0 wt% Cr, 3.5 wt% B, 4.5 wt% Si, bal. Ni) cladding coatings with different TaC additions (0 wt%, 5 wt%, 10 wt%, 20 wt%, 30 wt%, and 40 wt%) on a TC4 substrate by LC processing, which revealed that interdiffusion of substrate elements induced the formation of TiC, TiB, and TiB_2_, while the TaC addition suppressed these precipitations. Benefiting from the suppression effect, the coating with TaC addition had a higher TiO_2_ proportion in the passive film, compared with that without TaC addition. In addition, Ta_2_O_5_ also formed in the passive film, which contributed to the improvement of corrosion resistance as well. The increased TiO_2_ content cooperated with Ta_2_O_5_ in the passive film and improved the corrosion resistance of the Ni-based cladding coating. Zhang et al. [216] prepared the CoCrNi medium entropy alloy base coatings on TC4 substrates by LC assisted with ultrasonic vibration (UV), which demonstrated the BCC solid solution matrix strengthened with (Ni, Co)Ti_2_ and TiC was the main feature, as shown in Figure 11. The UV-assisting treatment could refine the precipitates and grain structure, which helps with the restricting of corrosion pits. In addition, the substructures were increased with the UV treatment, because of the redistribution of elements. Compared with the CoCrNi medium entropy alloy base coating fabricated by low laser energy, the coating by high laser energy demonstrated higher corrosion potential and corrosion impedance, which indicated a well improved corrosion resistance. The well optimized microstructure, precipitate, and elements distribution contributed the most to the improved corrosion resistance, especially the uniformly distributed corrosion elements. Therefore, a corrosion-resistant coating cooperated with the appropriate auxiliary technology would obtain the reduplicated corrosion performance.

Comparatively speaking, the phase with high chemical stability is very helpful to the corrosion-resistant coating, but the balance between the interfacial bonding and corrosion resistance should be considered systematically. In addition, the additive in the cladding coating plays an important role in the corrosion resistance, which enhances the corrosion resistance by the precipitation. For the corrosion-resistant coating fabricated by LC technology, the chemical composite, phase constituent, microstructure and bonding strength could influence the corrosion behavior of the cladding coating and should be considered synergistically.

### 4.3. High-Temperature Oxidation-Resistant Coatings

As a kind of material with high strength and low density, Ti alloys have been widely used in the aerospace industry as high-temperature components, such as compressor casings, rotor blades, etc. Though the in situ surface TiO_2_ oxide film could restrain oxidation, it is really thin and could not handicap the internal oxygen diffusion and inner oxidation. If the TiO_2_ oxide film on the Ti alloy surface is thick, the high interfacial stress would influence the bonding strength. For example, the Ti6Al4V alloy can work well below 350 °C, but it would experience serious oxidation and nitriding, when the service temperature is higher than 600 °C. This will lead to a sharp decline in the creep resistance and high-temperature oxidation resistance, which greatly limits their application at higher temperatures [217,218]. For Ti-based alloys, the “thermal barrier” protecting layer should be fabricated, if its service temperature exceeds the threshold of 600 °C [219]. Considering the lightweight of aerospace components, it is an issue to improve the oxidation resistance of the Ti alloys with a reasonable thickness of cladding coating.

**Figure 12:**
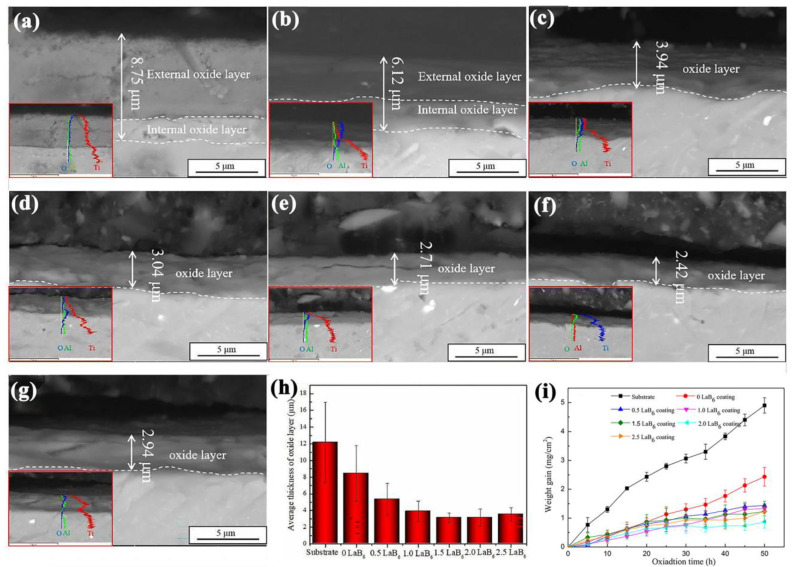
Cross-section morphology of oxide layers obtained at 600 °C for 50 h for the Ti-6Al-4V substrate and (TiC + TiBx)/Ti coatings with different amounts of LaB_6_ addition and corresponding weight gain curves with time: (**a**) Ti-6Al-4V substrate, (**b**) 0 wt% LaB_6_-doped coating, (**c**) 0.5 wt% LaB_6_-doped coating, (**d**) 1.0 wt% LaB_6_-doped coating, (**e**) 1.5 wt% LaB_6_-doped coating, (**f**) 2.0 wt% LaB_6_-doped coating, (**g**) 2.5 wt% LaB_6_-doped coating, (**h**) average thickness, (**i**) oxidation weight gain (Δw/A) curves (Reprinted from Ref. [193] with the kind permission of Elsevier).

Generally, the MCrAlY (M = Ni, Co, or NiCo) coatings have been considered as the most valuable thermal barrier coatings due to their excellent properties in oxidation resistance [220]. The well prepared α-Al_2_O_3_ + NiCoCrAlTaY bilayer coatings exhibit 6–10 times the oxidation resistance of the substrate, because of the low oxygen diffusivity and low growth rates [221]. In addition, their good adhesion to the alloy matrix and ceramics has made them an excellent transit layer. These advantages promote their wide application in the high-temperature protection coating. A recent study prepared a Ti-Cu-NiCoCrAlTaY composite coatings on the surface of a TC4 alloy by LC technology and revealed that the dense double-layer formed in the composite coating enhanced oxidation resistance further [222]. Guo et al. [8] prepared a NiCrBSi coating with and without WC addition on a TA2 pure titanium substrate by LC, which demonstrated that the presence of an intermetallic compound promoted the formation of dense and compact microstructures and benefited the oxidation resistance. In addition, A1_2_O_3_ could play an important role, because of its excellent oxidation resistance and high thermal stability under high-temperature conditions. Yin et al. [193] used LC processing to prepare the (TiC + TiBx)/Ti cladding coatings with different LaB_6_ additions (0 wt%, 0.5 wt%, 1.0 wt%, 2.0 wt%, and 2.5 wt%) on a TC4 substrates and investigated the effect of LaB_6_ addition on the high-temperature oxidation performance. At 600 °C for 50 h, the TC4 substrate underwent significant oxidation, but the (TiC + TiBx)/Ti cladding coating decreased the oxidation obviously, as shown in Figure 12. Figure 12a–g illustrates the cross-section morphology of the Ti-6Al-4V substrate and coatings with different amounts of LaB6 addition, and (h) shows the oxide layers’ average thickness. For the specimen with cladding coating, the addition of LaB_6_ decreased the oxidation weight gain from 2.43 mg/cm^2^ to 1.30 mg/cm^2^, when its content increased from 0 wt% to 1.0 wt%. Moreover, the oxidation law gradually changed from the linear law to the parabolic law when the LaB_6_ addition increased to 1.5 wt%, indicating that the oxide layer gradually became dense and protective, as shown in Figure 12i. Such an improvement should be attributed to the microstructure optimization by the LaB_6_ addition. Firstly, the rod-like TiB_2_ phase is refined by the added LaB_6_, reducing the crack sensitivity. Moreover, the addition of LaB_6_ refined the grain size of oxides and benefited the formation of a continuous and compact oxide layer. In addition, the in situ formed La_2_O_3_ lowers the oxygen content in the coating. Feng et al. [223] prepared a (Ti_3_Al + TiB/Ti) composite coating on TC4 by LC, which decreased the weight gain by 70%-80%. Compared with the TiO_2_ oxide layer on the bare TC4 surface, the (Ti_3_Al + TiB/Ti) composite coating formed a dense TiO_2_ + Al_2_O_3_ compound layer, and the Al_2_O_3_ layer could effectively hinder the diffusion of oxygen. In addition, the single TiO_2_ oxide layer generated high interface stress, which easily induced cracks in the interface of the coating, while the Al_2_O_3_-doped TiO_2_ compound layer decreased the stress concentration and the cracks, which is beneficial to the long-term service of the cladding coating. Similarly, Liu et al. [224] prepared a TiN/Ti_3_Al intermetallic composite coating on the surface of a TC4 alloy by LC and revealed that Al_2_O_3_ and TiO_2_ are the main formed phases during the isothermal oxidation at 600 °C and 800 °C. Due to the good balance between oxidation resistance and interface stress, the TiN/Ti_3_Al intermetallic composite coating demonstrated an excellent high-temperature protecting effect. Though the ceramic or intermetallic reinforced cladding coating could benefit the oxidation, the adhesion strength would be influenced. Especially for the component with high-temperature friction, the interaction of oxidation and friction requires the coating to possess the stiffness, oxidation resistance, and good adhesion simultaneously [225]. To achieve the requirement, the previous research added LaB_6_ to a Ti-TiC-TiBx composite coating prepared by LC and showed that the oxidation weight gain of the composite coating with LaB_6_ addition had been decreased to the half or less of that without LaB_6_ addition. In addition, the wear resistance of the composite coating was also improved obviously. Recently, a study tried to make full use of the Ti_5_Si_3_ to form TiO_2_ and SiO_2_ compound oxides on a Ti-based alloy, which revealed that the good oxidation resistance could be obtained at high temperature [226]. Such a feature of Ti_5_Si_3_ could be applied to fabricate the protection cladding coating on the Ti-based materials.

Based on the studies on the corrosion-resistant coating, it can be found that the regular metal-based coating could obtain a well bonded interface with the substrate, while the ceramic- or intermetallic-compound-reinforced composite coating would have better corrosion resistance. How to balance the bond strength and corrosion resistance is a crucial issue. The active elements, such as rare earth elements, have been thought as the effective one to improve the bond strength and corrosion resistance simultaneously. However, the appropriate addition amount should be fully evaluated with the corrosion resistance requirement. Therefore, the cladding coating which has integrated with the advantages of metallic and ceramic materials should be more prospective and have application valuable.

### 4.4. Biocompatible Coatings

As mentioned above, Ti-based materials have good biocompatibility, because of their high chemical stability. Compared with other metals, Ti-based materials are more favorable to be used in biomedical implants [170,172,173,175,227,228,229,230,231,232,233,234,235]. Due to the formation of TiO_2_ passivation film on their surface, Ti-based alloys have been defined as an inert metal and almost have no bioactivity, which influences tissue regeneration. Moreover, the ion dissolution from Ti-based alloys always induces an inflammatory reaction. To improve the bioactivity of a Ti-based alloy implant, surface modification should be performed, and LC has been considered as the most attractive one because of its well bonding strength. In addition, the chemical constituents of the surface modification layer could be more diversified based on the requirement of implants. Until now, bioactive ceramics such as hydroxyapatite (HA), fluorapatite (FA), and β-tricalcium phosphate (β-TCP) have been prepared on the Ti-based alloys’ surfaces by LC. Due to the similar chemical content or crystal structure to bones, these bioactive ceramics could play an important role in promoting osteogenesis and new bone generation [170].

**Figure 13:**
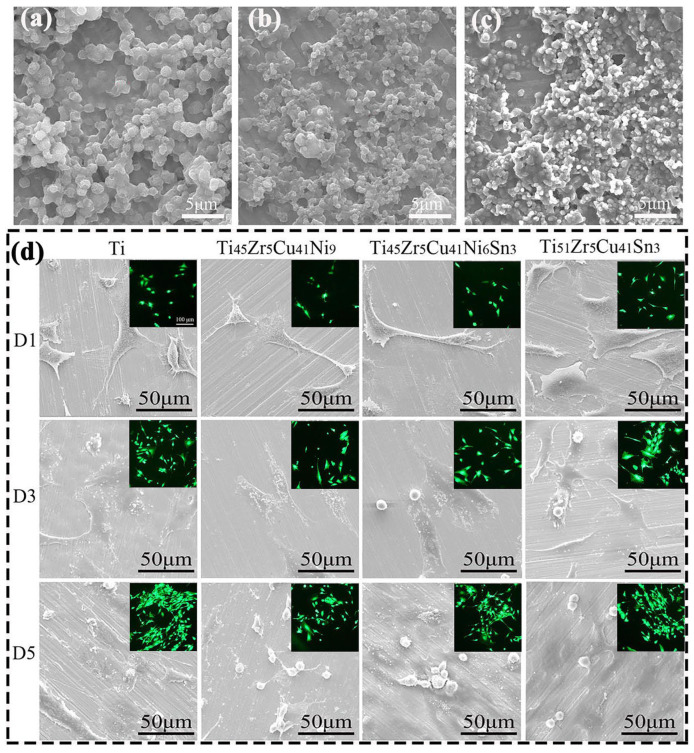
Surface morphology of a metallic glass composite coating immersed in SBF for 7 days: (**a**) Ti_45_Zr_5_Cu_41_Ni_9_ coating; (**b**) Ti_45_Zr_5_Cu_41_Ni_6_Sn_3_ coating; (**c**) Ti_51_Zr_5_Cu_41_Sn_3_ coating; (**d**) morphology of osteoblastic cells cultured on the substrate and metallic glass composite coatings for 1, 3 and 5 day (inset images showing the fluorescence micrograph and the live cells stained with green) (reprinted from Ref. [236] with the kind permission of Elsevier).

Due to the special crystal structure of HA, the high temperature during LC processing would lead to the decomposition of HA and the formation of unstable phases. Paital et al. [229] prepared an HA cladding coating on a TC4 alloy by LC and demonstrated that the phase constituents mainly include CaTiO_3_, TiO_2_, α-TCP, and Ca(OH)_2_, which could regenerate the thick HA layer in simulated body fluid (SBF) solution. The greatly improved wettability or hydrophilicity by HA promotes cell adhesion and proliferation, which is beneficial to osseointegration and osteogenesis. A previous study [231] prepared a Ca-P-based coating on a TC4 alloy substrate by LC, which demonstrated that LC processing changed the substrate surface to a coarser state with regular morphology. The evolved surface morphology and formed HA content induced the adhesion and proliferation of MC3T3-E1 osteoblast cells. In addition, the research added the copper in the Ca-Si-based coating fabricated on the TC4 alloy substrate by using LC technology and revealed the in situ formed Ca_2_SiO_4_, CaTiO_3_, and Cu_2_O phases inside [232]. Moreover, the compound phases promoted the formation of HA in the SBF solution and demonstrated the ultrafine squamous-like morphology. Due to the existence of Cu-based phases, the cladding coatings exhibited obvious antibacterial properties on E. coli, especially for the coating with higher Cu content. Such a feature could be applied to solve the bacterial infection problem during surgery. Additionally, the increased wettability by the HA benefited nutrition transfer, which promoted cell differentiation. Therefore, the biocompatibility of the TC4 alloy with a Ca-based coating was much better than that without coating. In order to improve the osteogenesis at the initial stage of implantation, Jiang et al. [233] doped antibiotics drugs in an HA coating, which could inhibit bacterial infection. Chakraborty et al. [234] prepared a titanium-based HA composite coating which almost matched the mechanical properties of human bones and had a better osteoinductive ability. The advantage of HA in drug carriers and its well-matched mechanical properties with bone promote its wide application in orthopedic clinics. Wu et al. [236] prepared three kinds of Ti-Cu-based metallic glass composite coatings (Ti_45_Zr_5_Cu_41_Ni_9_, Ti_45_Zr_5_Cu_41_Ni_6_Sn_3_, and Ti_51_Zr_5_Cu_41_Ni_3_) on a Ti substrate by LC, which could induce the deposition of calcium phosphate as shown in Figure 13a–c. Though the dissolution of the Cu ion could realize the antibacterial function, it also decreased the cytocompatibility. As shown in Figure 13d, the morphology of osteoblastic cells cultured on a substrate with metallic glass composite coatings for 1, 3, and 5 days (inset images showing the fluorescence micrograph and the live cells stained with green). It can be found that compared with the Ti substrate, the cell activity cultured on the Ti-Cu based metallic glass composite coatings decreased obviously, which indicated the cytotoxicity. Comparatively, the Ti_51_Zr_5_Cu_41_Sn_3_ coating showed better cytocompatibility, which could be developed further for the candidates of orthopedic implants and dental materials. This is attributed to relatively high amorphous content in the Ti_51_Zr_5_Cu_41_Sn_3_ coating.

Actually, FA has the same apatite phase as HA and shares a similar composition, while FA has the improved chemical stability, lower dissolution rate and better thermal stability. Thus, in order to avoid HA changing to an unstable phase at high-temperature, Chien et al. [227] used FA instead of HA and mixed it with 20 wt% α-Al_2_O_3_ to prepare the composite coating on a Ti6Al4V substrate by LC. The composite coating was mainly composed of FA, β-TCP, CaF_2_, θ-Al_2_O_3_, CaTiO_3_, and CaAl_2_O_4_ phases. It was found that after immersion in simulated body fluids for 14 days, a dense bone-like apatite layer was formed on the surface of the composite coating, which exhibited excellent biological activity. Wollastonite is another promising bioactive coating material for titanium alloys used in bone tissue repair and replacement, due to its osteoconductive properties [175]. In a CaO-SiO_2_-MgO ceramic composite coating prepared by LC, the silicon compound in the cladding layer can effectively promote the formation of apatite [172]. In addition, the FA layer was formed on the cladding layer surface, when the composite coating was immersed in simulated body fluid (SBF) for 21 days. The composite coating did not cause hemolysis and was non-toxic to cells, indicating its good biocompatibility. Zhu et al. [173] also prepared calcium phosphate bio-ceramic coatings containing bioactive phases of β-TCP and HA and revealed that the osteoclast precursors on the coating have excellent proliferation ability. Furthermore, the coating can be digested by osteoclasts and have excellent biological activity.

Due to the special physical and mechanical properties of the Ti-based alloys, they were widely applied in the orthopedic implant. To improve the osteogenesis and osseointegration capability of the Ti-based alloy implant, the bioactive coatings such as HA, FA, and β-TCP demonstrate obvious advantages, due to the bone induction effect. The similar crystal structure and chemical composition of these bioactive coatings benefit the adhesion and differentiation of osteoblasts. However, due to the complex in vivo physiological environments, the biological coating fabricated by LC still needs further verification in clinical trials.

### 4.5. Other Functional Coatings

**Figure 14:**
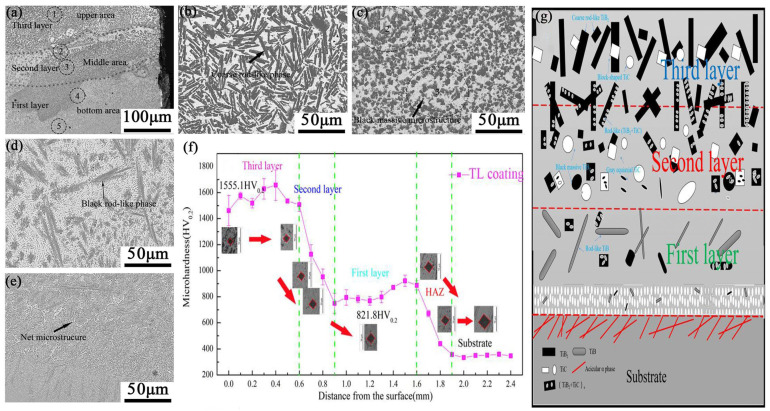
Microstructure, microhardness, and schematic diagrams of TiNi/Ti_2_Ni-based composite coatings with gradient distribution of TiC- and TiBx-reinforced phases on the TC4 alloy. (**a**) Overall microstructure along the cross-sectional direction, (**b**) enlarged microstructure of area 1 in (**a**), (**c**) enlarged microstructure of area 2 and 3 in (**a**), (**d**) enlarged microstructure of area 4 in (**a**), (**e**) enlarged microstructure of area 5 in (**a**), (**f**) microhardness variation along the cross-sectional direction, and (**g**) schematic diagram showing the reinforced phases distribution (the red line indicates the demarcation line of each coating) (reprinted from Ref. [241] with the kind permission of Elsevier).

Besides wear-resistant coatings, corrosion-resistant coatings, high-temperature anti-oxidation coatings, and biological coatings, some studies have developed the multi-functional or gradient functional coatings by using LC technology. For example, protection coatings with a high proportion of stiffness phases always have high hardness but low ductility, which is prone to inducing microcracks during their service and restrains their application. In recent years, gradient functional coatings have been developed and exhibit the gradient evolution from substrate to surface in microstructure, phase constituent, and mechanical properties [237]. With such a gradient evolution, the stress concentration could be well eliminated and the service life can be improved greatly [238]. Cui et al. [239] prepared FeCoCrNiMnAl_0.5_-FeCoCrNiMnAl gradient HEA coatings with a body-centered cubic crystal layer and a face-centered cubic crystal layer, which exhibited the dynamic recrystallization and grain deformation in different layers. Such a gradient function layer structure could not only relieve the piling up of dislocations but also refine the superficial microstructure, which contributes to the toughness and strength simultaneously. Lin et al. [240] prepared a TiB_2_/TiB gradient coating on a TC4 alloy by LC, which made full use of the distribution of TiB_2_/TiB to increase the hardness and restrict the initiation of cracks. Liang et al. [241] prepared TiNi/Ti_2_Ni-based composite coatings with a gradient distribution of TiC- and TiB_x_-reinforced phases on the TC4 alloy, which demonstrated the typical gradually decreased microhardness from surface to substrate, as shown in Figure 14f.

Figure 14a–e illustrates the microstructures of feature areas in the triple-layer coating: (a) is the overall microstructure of the triple-layer coating; (b–e) are the upper area (TL coating), transition region between the upper area and middle area, the middle area, the transition region between the middle area and bottom area, and the bottom area in the triple-layer coating, respectively. From these pictures, it can be seen that all feature areas show different microstructures. Different microstructures exhibit different microhardness values (Figure 14g). The reason is that the content of ceramics is high, but the gradient distribution feature well relieves the stress concentration and eliminates the microcracks. Due to the synergistic strengthening effect of the triple layers, the composite coating exhibited excellent wear resistance. 

Actually, the service condition for a Ti-based alloy component is always complex. Especially for a component working in extreme environments, the friction would be accompanied by vibration. The vibration would result in the irregular load on the friction pairs, which deteriorates the wear resistance and decreases the service life [242]. To solve the problem, solid lubricating phases are added to the cladding material to realize the self-lubricating function which relieves the excessive wear by overload. Shakti et al. [243] prepared a Ni-WS_2_-Ti-6Al-4V self-lubricating coating on a TC4 alloy and revealed that the lubricate phase WS_2_ could be well dispersed in the cladding coating, which decreased the wear lost to half of the substrate and the friction coefficient to 0.25, because the adhesion wear became the predominant mechanism. Liu et al. [244] prepared a TiN/WS_2_ + hBN/NiCrBSi composite coating with WS_2_ and TiS lubricant phases on a TC4 alloy, which realized the self-lubricating effect and ultra-high microhardness. The friction behavior exhibited that the composite coating decreased the friction coefficient to about 0.3458 and the wear rate obviously.

Other coatings with multi-functions are also continually developed to meet work environment requirements. SELVAN J.S [245] prepared a Ni-SiC-based high-temperature thermal barrier coating on pure Ti by LC, which possessed TiNiSi, TiSi, Ti_5_Si_3_, and NiTi_2_ phases with high thermal and chemical stability. The intermetallic-compound-strengthened composite coating exhibited the balanced properties and met the requirement of working in thermal, mechanical, corrosive, and erosive environments. Based on the laser cladding technology, nitriding was performed on the Ti-5Al-5Mo-5V-1Cr-1Fe coating and formed a TiN-reinforced phase in the coating, which improved the fretting resistance obviously [246]. Gushchina [247] fabricated an alternating Ti6Al4V and Cp-Ti multilayer coating by LC to enhance the impact strength of Cp-Ti. Compared with the crack propagation energy of the Ti substrate (90.5 ± 3.9 J, 91.8 ± 0.3 J and 87.9 ± 8.3 J), those of the specimens with multilayer coating were decreased to half or less (31.1 ± 2.3 J, 49.8 ± 6.3 J and 37.1 ± 7.0 J) in different directions. The improved crack resistance should be attributed to the Ti6Al4V layer which is the main contributor to anisotropy of the fracture energy. Generally, the development of functional cladding coatings is mainly based on the working environment requirement, while their properties are dependent on the microstructure design and phase constituent.

**Figure 15:**
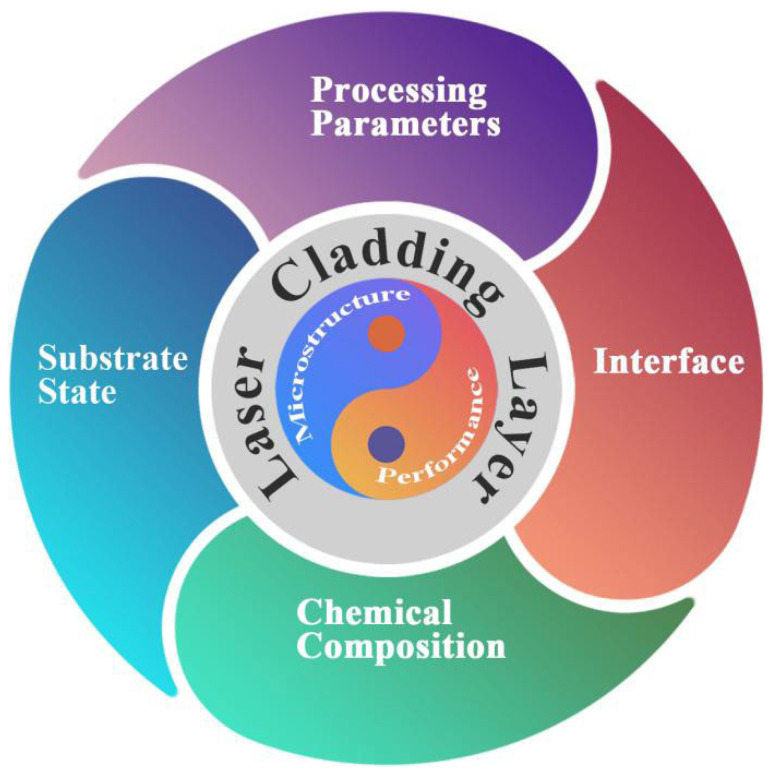
The schematic diagram of the main influencing factors on the laser cladding coating.

According to the above studies on surface modification of Ti and its alloys by LC, it can be concluded that the design and fabrication of the coatings are mainly dependent on the requirement of the working or service environment. The properties are the crucial factor for the coating, whether it is single-phase or multi-phase. However, the microstructure and properties are closely related to each other [58,247]. A well-optimized microstructure would enhance the mechanical properties, corrosion resistance, wear resistance, oxidation resistance, biocompatibility, and so on. Though the in situ precipitation or extrinsic addition of ceramic, intermetallic compound particles would strengthen the cladding coating obviously, the balance between brittleness and ductility is still a critical issue. Considering the functional requirement, the surface modification of Ti and its alloys should be designed systematically according to the schematic diagram shown in Figure 15. Before the cladding coating fabrication, the chemical composition or phase constituent should be designed based on the working environment requirements and substrate properties. Furthermore, the reasonable active element addition would improve the distribution of the strengthening phase and the phase interface. After that, the processing parameters of LC should be well optimized to eliminate metallurgical defects and obtain the ideal microstructure. Moreover, the interface between the cladding coating and substrate should be well treated to enhance the interface bonding strength. The interface including the phase interface, layer interface, and substrate interface plays an important role in microstructure stability, thermal stability, chemical stability, and mechanical reliability. Therefore, the substrate state, chemical composition of the cladding coating and substrate, the processing parameters, and the interface comprise the critical factors which influence the microstructure and properties of the cladding coating prepared by LC. How to systematically optimize these factors and obtain well-balanced performance are long-term research issues. The in-depth investigation on these factors would explore the inner mechanism and develop more protective coating or technology for Ti and its alloys, which helps to extend the application field of this kind of materials.

## 5. Conclusions

Ti and its alloys have been widely applied in many fields, because of their fascinating advantages in their mechanical properties, corrosion resistance, biocompatibility, and so on. However, Ti and its alloys face many challenges, if their components work in more severe or complex environments. Generally, the surface is always the origin of failure for Ti and its alloy workpiece, which influences the performance degradation and service life. To improve the properties and function, surface modification becomes the common processing for Ti and its alloys. In the present work, the technology and development of laser-cladding-modified Ti and its alloys have been reviewed, according to the cladding technology, cladding materials, and coating function.

The laser cladding parameters could influence the temperature distribution and elements diffusion in the molten pool by the amount and efficiency of input energy, which changes the quality and properties of the laser cladding coating. Auxiliary technology improves the elements’ transfer and temperature distribution in the molten pool through the external mechanical energy, electric, or magnetic field, which benefits microstructure optimization and properties improvement. The matrix of the laser cladding coating determines the basic properties and should be well designed based on the requirements of the service environment, while the reinforced phases or particles, such as ceramic and intermetallic compounds play an important role in the laser cladding coating, which could increase the hardness, strength, wear resistance, oxidation resistance, corrosion resistance, biocompatibility, and so on. However, the excessive addition of a reinforced phase or particles would deteriorate the ductility, and thus the balance between functional properties and basic properties should be considered during the design of the chemical composition of laser cladding coatings. In addition, the interface including the phase interface, layer interface, and substrate interface plays an important role in microstructure stability, thermal stability, chemical stability, and mechanical reliability. Therefore, the substrate state, the chemical composition of the laser cladding coating and substrate, the processing parameters, and the interface comprise the critical factors which influence the microstructure and properties of the laser cladding coating prepared.

In the future, a laser cladding coating with better properties would still be the main research focus. Especially for the workpiece serviced in harsh environments, the Ti and its alloys with an excellent surface cladding coating would be more prospective, because of its good adhesion with the substrate. To realize this goal, well designed cladding coating structure and high-precision manufacturing technology should be developed firstly. Then, the integration of multi-phase strengthening and a reasonable layer structure would be one of most promising research topics, which would produce the multi-functional coating, but the precise regulation on the phase distribution is a challenge. Moreover, how to make full use of auxiliary technology to optimize the microstructure of the cladding coating would be a research focus, which could improve the properties further. In general, the development of a laser cladding coating on Ti and its alloys would mainly depend on the requirements of their structural and functional components. How to systematically optimize the influencing factors and obtain well-balanced performance are long-term research issues.

## Figures and Tables

**Table 1:** The characteristics of the different methods.

Characteristic	BW	AC	PC	EBC	LC
Energy density	Low	Low	Moderate	Moderate	High
Automaticity	Low	Moderate	High	High	High
Heat-affected zone	High	High	Moderate	Moderate	Low
Controllability	Low	Moderate	High	High	High
Cost	Low	Moderate	Moderate	Moderate	High

**Table 2:** The self-fluxing alloy powders for the surface modification of Ti-based alloys by LC.

Cladding Materials	Substrates	Strengthening Phases	Properties	Ref.
Co42 + TiN	TC4 Alloy	TiN + TiC + Co_3_Ti + TiC_0.3_N_0.7_ + NiTi	H + WR	[107]
Co42 + B_4_C + SiC + Y_2_O_3_	TC4 Alloy	CoTi + CoTi_2_ + NiTi + TiC + TiB_2_ + TiB + Cr_7_C_3_ + Ti_5_Si_3_	H + WR	[105]
Co50	TA15	TiB_2_ + Cr_5_Si_3_ + TiC + SiC + Co_3_Ti + NiC + WB	H + WR	[108]
Co42 + SiC	TC4 Alloy	CoTi + CoTi_2_ + NiTi + Cr_7_C_3_ + TiB + TiC + Ti_5_Si_3_	H + WR	[109]
NiCrBSi + WC	TA2	Cr_2_Ni_3_ + Cr_3_Si + TiC + WC + W_2_C + B_4_CrTi	H + WR + OB	[8]
NiCrBSi+ B_4_C	TC4 Alloy	γ-Ni + TiB_2_ + TiC + CrB	H	[110]
NiCrBSi + TiC	TC4 Alloy	TiC + Cr_23_C_6_ + CrB + TiB_2_	WR	[111]
NiCrMoNb	α-Ti Alloy	Cr_23_C_6_ + Cr_5_B_3_ + NbC	H + WR	[100]
NiCrBSiC		Cr_23_C_6_ + Cr_5_B_3_ + CrB	H + WR	
Ni45 + TC4 + NiCr-Cr_3_C_2_	TC4 Alloy	TiC + TiB_2_ + Ti_2_Ni	H + WR	[112]

Remarks: hardness (H); wear resistance (WR); biocompatibility (B); corrosion performance (CP); oxidation behaviors (OB).

**Table 3:** The HEA powders for the surface modification of Ti-based alloys by LC.

Cladding Materials	Substrates	Strengthening Phases	Properties	Ref.
FeNiCrMoWSiBC	TA2	Fe_2_Ti + Fe_2_B + Fe_3_Si + Ti_2_Ni	H + WR	[118]
CoCrFeNiNb	Pure Ti	BCC + Cr_2_Ti+ Cr_2_Nb	H	[119]
AlTiVNbMo	TC4 Alloy	BCC	H	[120]
CoCrFeNiVTi	TC4 Alloy	BCC + (Co,Ni)Ti_2_	H + WR	[121]
TiAlNiSiV	TC4 Alloy	BCC + (Ti,V)_5_Si_3_ + TiN	H + WR	[122]
NiCrCoTiVAl	TC4 Alloy	BCC + FCC	H	[123]
TiVCrAlSi	TC4 Alloy	BCC + (Ti,V)_5_Si_3_	H + WR	[124]
TiVCrAlSi	TC4 Alloy	BCC + (Ti,V)_5_Si_3_	H + WR + OB	[125]
FeCoCrNi	TC4 Alloy	FCC + BCC + Cr_7_C_3_	H + CP + OB	[126]
AlCrNiSiTi	Ti64 Alloy	(Ti,Cr)_5_Si_3_ + NiAl	H+ WR	[127]
NiCrCoTiV	TC4 Alloy	BCC + (Ni,Co)Ti_2_	H + WR	[128]
AlNbMoTaCu	TC4 Alloy	HCP + FCC + BCC	H + WR	[129]
NbMoTaWTi	TC4 Alloy	BCC	H+WR	[130]
AlBCoCrNiTi	TC4 Alloy	BCC + TiB_2_ + (Co,Ni)Ti_2_	H + WR	[131]
AlCoCrFeNiTi	TC4 Alloy	BCC + TiN + (Ni,Co)Ti_2_	CP	[132]
AlCoCrCuFeNi	TC4 Alloy	BCC	H + WR	[133]

Remarks: hardness (H); wear resistance (WR); biocompatibility (B); corrosion performance (CP); oxidation behaviors (OB).

**Table 4:** The ceramic/metal composite powders for the surface modification of Ti-based alloys by LC.

Composite Material Systems		Substrates	Reinforced Phase	Properties	Ref.
Ceramics	Metals				
B_4_C	NiCrBSi	TC4 alloy	TiC + TiB_2_ + CrB	H	[110]
B_4_C	Ti + Ni	TA15 alloy	TiB + TiC + TiNi + Ti_2_Ni	H and WR	[144]
B_4_C	Ti811	Ti811 alloy	TiC + TiB	H	[145]
B_4_C	TC4	TC4 alloy	TiC + TiB_2_ + TiB	H and WR	[146]
B_4_C	Ni60A	TC4 alloy	TiC + TiB_2_ + CrB + Ni_3_Ti	H and WR	[147]
TiC + B_4_C	Ni204	TC4 alloy	TiC + TiB_2_	H and WR	[61]
TiC	Ti	TC4 alloy	TiC	H	[148]
TiC	NiCrBSi	TC4 alloy	TiC + TiB_2_	H	[149]
TiC	Ti-Ni-Si	TA15 alloy	TiC	H and WR	[150]
TiC	NiCrBSi	TC4 alloy	Cr_23_C_6_ + TiC + TiB_2_ + CrB	WR	[111]
TiC	Ti + Al + Si	TC4 alloy	TiC + TiAl_3_	H and WR	[151]
TiC	Al	TC4 alloy	Ti_3_Al + Al_3_Ti + TiAl + TiC	H	[152]
TiC + TiB_2_	Ti	TC2 alloy	TiC + TiB_2_	H and CP	[153]
TiB_2_	TC4	TC4 alloy	TiB_2_ + TiB	H and WR	[154]
TiB_2_	Ti	TC4 alloy	TiAl_3_ + TiAl + Ti_3_Al + TiB_2_	H	[155]
TiB_2_	Ti	TC4 alloy	TiB +TiB_2_ + B27	H and WR	[156]
TiB_2_ + Al_2_O_3_	Fe_3_Al	TC4 alloy	Ti_3_Al + Fe_3_Al + TiB_2_ + Al_2_O_3_	H	[157]
TiB_2_	TC4	TC4 alloy	TiB_2_ + TiB	H and WR	[158]
TiN	Al	TC4 alloy	TiN + Ti_3_Al + TiAl + Al_3_Ti	H and WR	[159]
TiN	Co42	TC4 alloy	NiTi + TiN + TiC + TiB	H and WR	[107]
TiN	Ti + Al	TC21 alloy	Ti_3_Al + Ti_3_AlN + TiN	H and WR	[160]
TiCN	Ti	TC4 alloy	TiCN + TiO_2_	H and WR	[161]
h-BN	TC4	TC4 alloy	TiN + TiB+ Ti_3_N_1.29_ + BN	WR and B	[162]
h-BN	Ti	Ti-3Al-2V alloy	TiN + TiB + BN	H and WR	[163]
TaC	NiCrBSi	TC4 alloy	TiC + TiB_2_ + TiB + TaC	OB	[164]
WC	Ni60A	TA2	WC + TiC	H, OB, and WR	[8]
WC	Co + Ti	TC4 alloy	TiC + TiB_2_ + Cr_3_C_2_ + WC	WR	[165]
WC	TC4	TC4 alloy	W + WC + W_2_C + TiC	H	[166]

Remarks: hardness (H); wear resistance (WR); biocompatibility (B); corrosion performance (CP); oxidation behaviors (OB); tensile properties (TP); geometric properties (GP); magnetic properties (MP).

**Table 5:** The rare-earth-doped powders for the surface modification of Ti-based alloys by LC.

Cladding Materials	Substrates	Strengthening Phases	Properties	Ref.
TC4 + NiCr-Cr_3_C_2_ + CeO_2_	TC4 Alloy	FCC + CrTi_4_ + TiC	H	[68]
Ni25 + CeO_2_	TC4 Alloy	Ti_2_Ni + Ni_3_Ti + TiC	H + CP	[184]
FeCoNiCrMo + CeO_2_	TC4 Alloy	BCC + FCC	H + CP	[185]
TC4 + NiCr-Cr_3_C_2_ + CeO_2_	TC4 Alloy	FCC + CrTi_4_ + TiC	H + WR	[186]
Ni60 + TiN + CeO_2_	TC4 Alloy	Ti(C,N) + Ni_3_Ti + Cr_7_C_3_ + TiC	H + WR	[187]
Co42 + B_4_C + CeO_2_	TC4 Alloy	CoTi_2_ +NiTi + TiC + Cr_7_C_3_ + TiB_2_ + TiB	H + WR	[188]
TiC + ZrO_2_ + CeO_2_	TC4 Alloy	TiC + TiO + VC + TiVC_2_	H + WR	[187]
TiCN + SiO_2_ + CeO_2_	TC4 Alloy	TiN + Ti_6_O + Ti_3_SiC_2_	H + WR	[189]
TC4-Ni60-CeO_2_	Ti811 Alloy	TiC + Ti_2_Ni + TiB_2_	H + WR	[190]
Ni60A + CeO_2_	TC4 Alloy	Ti_2_Ni + TiC + TiB_2_	H + WR	[182]
Ni60-TiN-C-CeO_2_	TC4 Alloy	TiN+ Ti(C,N)+ TiC	H + WR	[191]
TC4 + B_4_C + LaB_6_	TC4 Alloy	TiC + TiB + TiB_2_	H + WR	[146]
Ti + AlB_2_ + LaB_6_	TC4 Alloy	TiB + Ti_3_Al	H + WR	[192]
TC4 + B_4_C + LaB_6_	TC4 Alloy	TiO_2_ + TiC + TiB + TiB_2_	OB	[193]
NiCrBSi + WC + Y_2_O_3_	TC4 Alloy	TiC + TiB_2_ + Ni_3_B	H + WR	[194]
TC4 + NiCr-Cr_3_C_2_ + Y_2_O_3_	TC4 Alloy	FCC + CrTi_4_ + TiC	H + WR	[195]
Ti + h-BN + Y_2_O_3_	TC4 Alloy	TiB + TiN	H + WR	[196]
Ti + B_4_C + Al + Y_2_O_3_	TC4 Alloy	TiB + TiC	H + WR	[197]
Co42-B_4_C-SiC-Y_2_O_3_	TC4 Alloy	CoTi + CoTi_2_ + NiTi + TiC + TiB_2_ + TiB + Cr_7_C_3_ + Ti_5_Si_3_	H + WR	[105]

Remarks: hardness (H); wear resistance (WR); biocompatibility (B); corrosion performance (CP); oxidation behaviors (OB).

## Data Availability

Not applicable.
